# A Kinetic Photometric Assay for the Quantification of the Open‐Chain Content of Aldoses

**DOI:** 10.1002/ejoc.202001641

**Published:** 2021-04-08

**Authors:** Hubert Kalaus, Alexander Reichetseder, Verena Scheibelreiter, Florian Rudroff, Christian Stanetty, Marko D. Mihovilovic

**Affiliations:** ^1^ Institute of Applied Synthetic Chemistry TU Wien Getreidemarkt 9 1060 Vienna Austria; ^2^ Department of Pharmaceutical Chemistry University of Vienna Althanstraße 14 1090 Vienna Austria

**Keywords:** Aldehydes, Analytical methods, Carbohydrates, Kinetics, UV/Vis spectroscopy

## Abstract

Aldoses exist predominantly in the cyclic hemiacetal form, which is in equilibrium with the open‐chain aldehyde form. The small aldehyde content hampers reactivity when chemistry addresses the carbonyl moiety. This low concentration of the available aldehyde is generally difficult to ascertain. Herein, we demonstrate a new kinetic determination of the (minute) open‐chain content (OCC) of aldoses. This kinetic approach exploits the aldehyde‐selectivity of 2‐aminobenzamidoxime (ABAO), which furnishes a strongly UV‐active adduct. Simple formation curves can be measured in a photometer or plate reader for high‐throughput screening. Under pseudo‐first order kinetics, these curves correlate with a prediction model yielding the relative OCC. The OCCs of all parent aldoses (pentoses and hexoses) were determined referencing against the two tetroses with exceptionally high OCCs and were in very good agreement with literature data. Additionally, the assay was extended towards higher‐carbon sugars with unknown OCC and also applied to rationalise a lack of reactivity observed in a recent synthetic investigation.

## Graphical abstract

An operationally simple UV‐based kinetic assay allows the accurate determination of the minute proportions (<1 %) of the open‐chain forms (OCC) of aldoses in equilibrium with the dominant ring forms. The OCC‐data of all parent aldoses has been determined with this new ABAO‐assay and is fully consistent with the literature gold standard, allowing extension into first practical synthetic challenges.

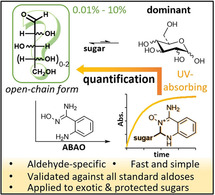

Carbohydrates are the predominant class of biomolecules formed by Nature, fulfilling a multitude of purposes in all living organisms: polysaccharides serve as structural components of cells, oligosaccharides play a major role in the recognition process of biomolecules, and monosaccharides and lower oligosaccharides are essential energy sources within metabolic cycles.^[1]^ Chemically, each monosaccharide comprises a carbonyl group (ketone or aldehyde) and a varying number of alkyl hydroxyl groups. Owing to these structural features, the open‐chain form of carbohydrates is in equilibrium with hemiacetal‐ring forms, which are generally dominating by several orders of magnitude with typical open‐chain contents (OCC) of significantly below 1 %.

Nonetheless, utilising the aldehyde moiety represents a powerful and low‐threshold opportunity for the more general organic chemist to exploit carbohydrates as a renewable source of chirality in synthetic strategies. Established methodologies include Wittig‐type olefinations,^[2]^ addition of organometallics (Mg, In, Zn)^[3]^ or carbanions (Kiliani‐Fischer), and Carbonyl‐Umpolung as principle examples^[4]^ (Figure 1, top). Despite its intrinsic reactivity, the aldoses’ aldehyde moiety is underutilised in both the field of carbohydrate chemistry and beyond.^[5]^

**Figure 1:**
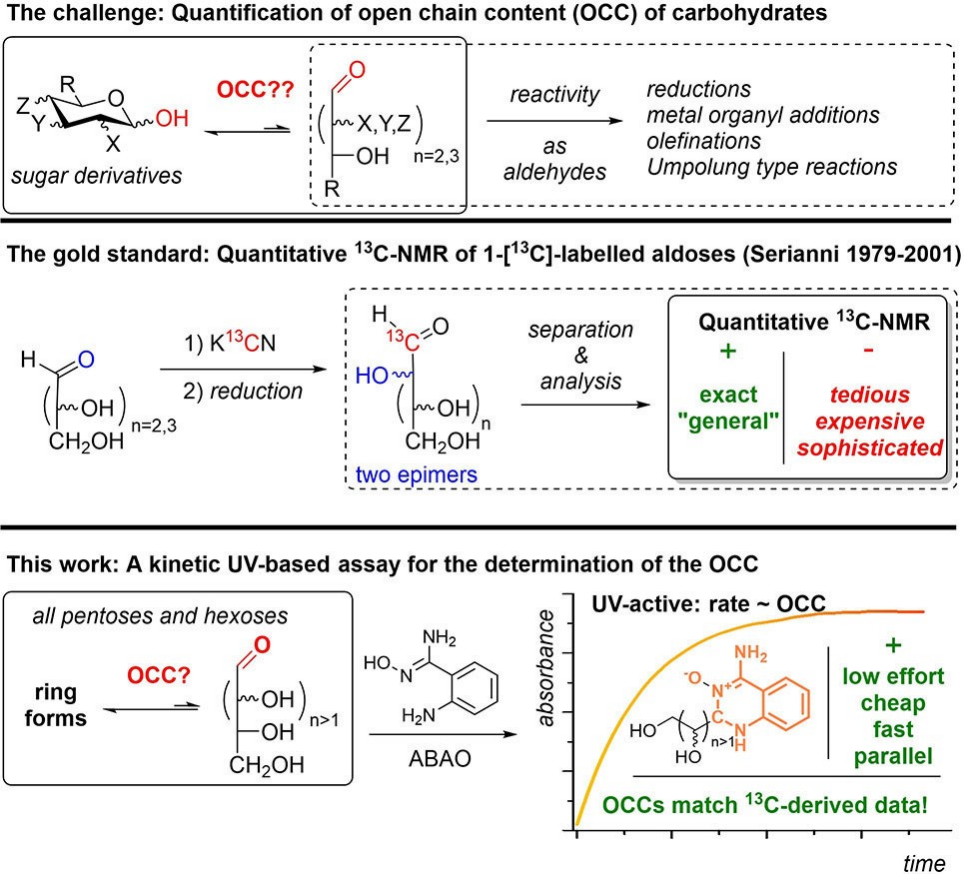
The open‐chain content (OCC) of carbohydrates as a relevant feature for reactions addressing their aldehyde moiety. The previous gold standard of OCC determination and our new operationally simple UV‐based approach.

We are convinced that the low OCC, particularly of the more popular monosaccharides, together with the difficulty to accurately determine the degree of availability (OCC), is an additional challenge to such studies. It prevents a separate discussion/analysis of the actual chemistry of interest from the influence of this pre‐equilibrium when comparing different carbohydrate species. We have observed examples for the dominating influence of the OCC in our own work with the indium mediated acyloxyallylation (IMA)[^3d^, ^6^] as well as NHC‐catalysed dehomologation.^[7]^ Also, in other reports, similar differences can be deduced although rather implicitly reported.[^5c^, ^8^]

We believe that the lack of a straightforward OCC determination is the reason why this important phenomenon is often not fully addressed, thereby representing a burden for accurate prediction and planning of synthetic outcome, creating a bias against the use of carbohydrates for this purpose.

To date, the quantification of the minor component open‐chain form, which is often present below 0.1 %, is challenging, being reflected in largely fluctuating values reported for the few carbohydrates for which such data exists at all.^[9]^ The currently available methods include circular dichroism,^[10]^ a kinetic NMR‐based assay,^[11]^ or the quantification by ^13^C‐NMR of 1‐[^13^C]‐labelled monosaccharides by the group of Serianni. The latter approach is providing the most convincing data but relies on the preparation of the monosaccharide of interest in its isotopically labelled form, representing a major limitation for broad and facile application^[12]^ (Figure 1, middle). A simpler to use approach to quantify the availability of certain aldoses as aldehyde species is required.

Herein, we report an efficient, facile, and reliable way to determine OCC values based on a UV‐based kinetic assay utilising the recently developed aldehyde‐selective reagent, 2‐aminobenzamidoxime **2** (ABAO, Figure 1, bottom) from the Derda group.^[13]^ We hypothesised that by shifting the equilibrium from closed to open forms, carbohydrates could be fully converted to ABAO‐adducts and that this formation can be easily followed due to the strong UV‐absorption (Figure 2a) of the ABAO adducts. We further assumed that the derived rate of formation could be correlated to the OCC. Hence, carbohydrates with a higher OCC exhibit faster adduct formation as is exemplified in Figure 2b with idose, arabinose, and galactose with decreasing known OCC. Herein, we show the validity of this approach, applying the assay to determine the OCC of all standard aldoses, validated them against the literature, and finally present its application in two recent synthetic challenges.

**Figure 2:**
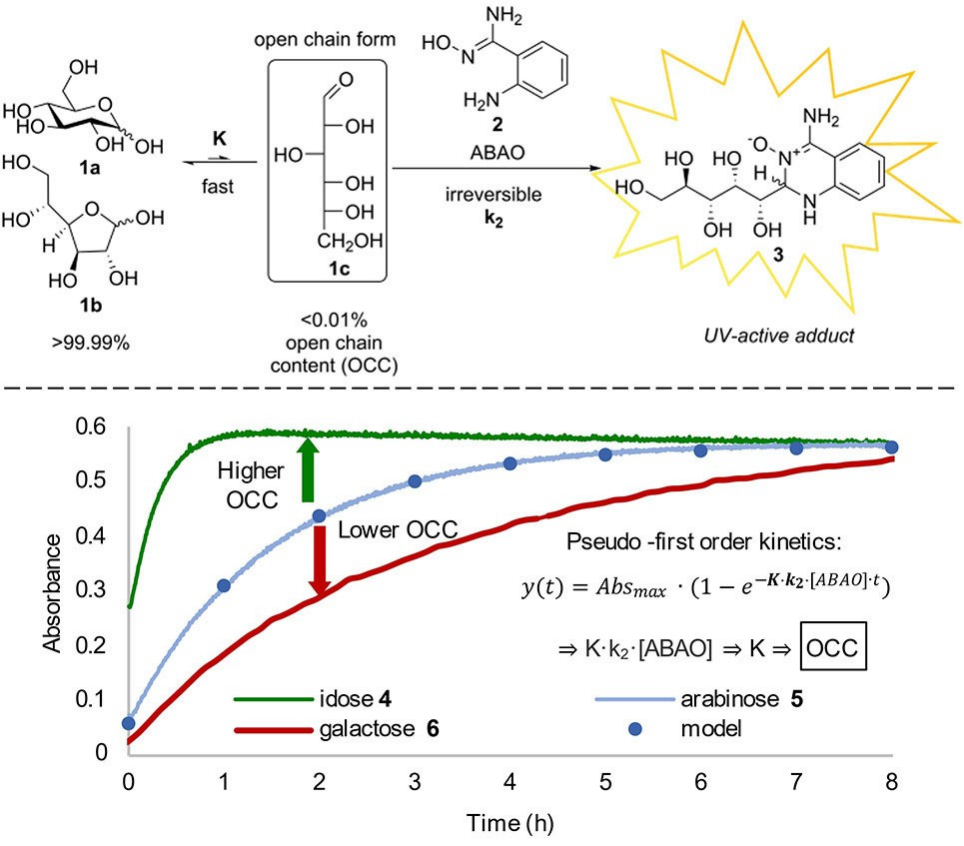
The kinetic aldehyde‐selective ABAO assay enables quantification of open‐chain contents (OCC) of aldoses. (a) The overall reaction leading to the UV‐active adduct that is detected. (b) The principal concept for the correlation between observed reaction rate and OCC.

The experimental setup of the assay is simple. Aqueous solutions of the respective aldoses are pipetted into a buffered solution (pH 4.5) of the ABAO component. Generally, the monosaccharide concentration was adjusted to 4 mM to ensure a sufficient reaction rate to also enable the investigation of monosaccharides with very low OCC. A tenfold excess of ABAO was applied to ensure pseudo‐first order kinetic condition, required for facile analysis. Another prerequisite for the approach is that the adduct formation and not the pre‐equilibrium is the rate‐determining step; this was proven experimentally to be clearly the case even with the slowest investigated monosaccharide, glucose **1** (see ESI). The structure of the postulated adducts, as well as their complete formation, was confirmed by NMR (see ESI for selected examples of the processed adducts).

The reaction progress is monitored by determination of the UV‐signal at 405 nm. The assay can be performed in a photometer cuvette or in multi‐well plates. Under pseudo‐first order conditions, the absorption curves follow a shape fitting the mathematical formula, equation 1:

(1) $$Abs={Abs}_{max}\cdot \left(1-e^{-\alpha \cdot t}\right)$$

Under the given assumptions and based on mathematical derivation which is found in the ESI, the exponent α reflects equation 2:

(2) $$\alpha =K\cdot k_{2}\cdot \left[ABAO\right]$$

and was obtained from software supported fitting or alternatively least‐square fitting of the logarithmic plot of (1–Abs/Abs_max_) against time. Herein, K is the equilibrium constant between all open‐chain species (Figure 2a) and k_2_ the rate constant of adduct formation. With known and constant ABAO concentration, the mathematical product K ⋅ k_2_ is deduced, interlinking the existing pre‐equilibrium (of interest) with the adduct formation (Figure 2b).

Thus, with known k_2_, absolute values of K (and OCC) can be deduced and the other way around. Therefore, at least one reference carbohydrate with a reliable OCC value is required.

Among the parent aldoses, the two tetroses erythrose **7** and threose **8** stand out with their extremely high OCCs of ∼10 %. Therefore, these were selected as potential reference points, and OCCs were conveniently quantified by ^1^H‐NMR (ESI). The assessment of erythrose **7** and threose **8** in the ABAO assay led to the unexpected finding that despite comparable OCCs, significantly differing slopes, reflecting different k_2_ values, were found. Additionally, different absorption maxima were observed (see Figure 3a) for the curves. This ambivalent behaviour between 2,3‐*erythro* and 2,3‐*threo* configured monosaccharides was consistently observed within the pentoses (Figure 3b) and hexoses (Figure 3c and Figure 3d) in the course of the study. The differences in the absorption maxima are attributed to different ratios of the two epimers formed during adduct formation within the two families, under the assumption of different UV‐absorption coefficients of those epimers. This interpretation is supported by the performed NMR‐measurements (ESI). As for the different slopes of the curves of formation, we concluded that the *erythro* configuration of the 2*O*,3*O*‐diol significantly favours product formation over the 2O,3O‐*threo*‐configuration. Therefore, as a refinement of our initial hypothesis, we decided to determine separate k_2, erythro_, and k_2, threo_ values for the 2,3‐*erythro* and the 2,3‐*threo*‐family, respectively (see ESI for a visual representation of those families in their Fischer projection).

**Figure 3:**
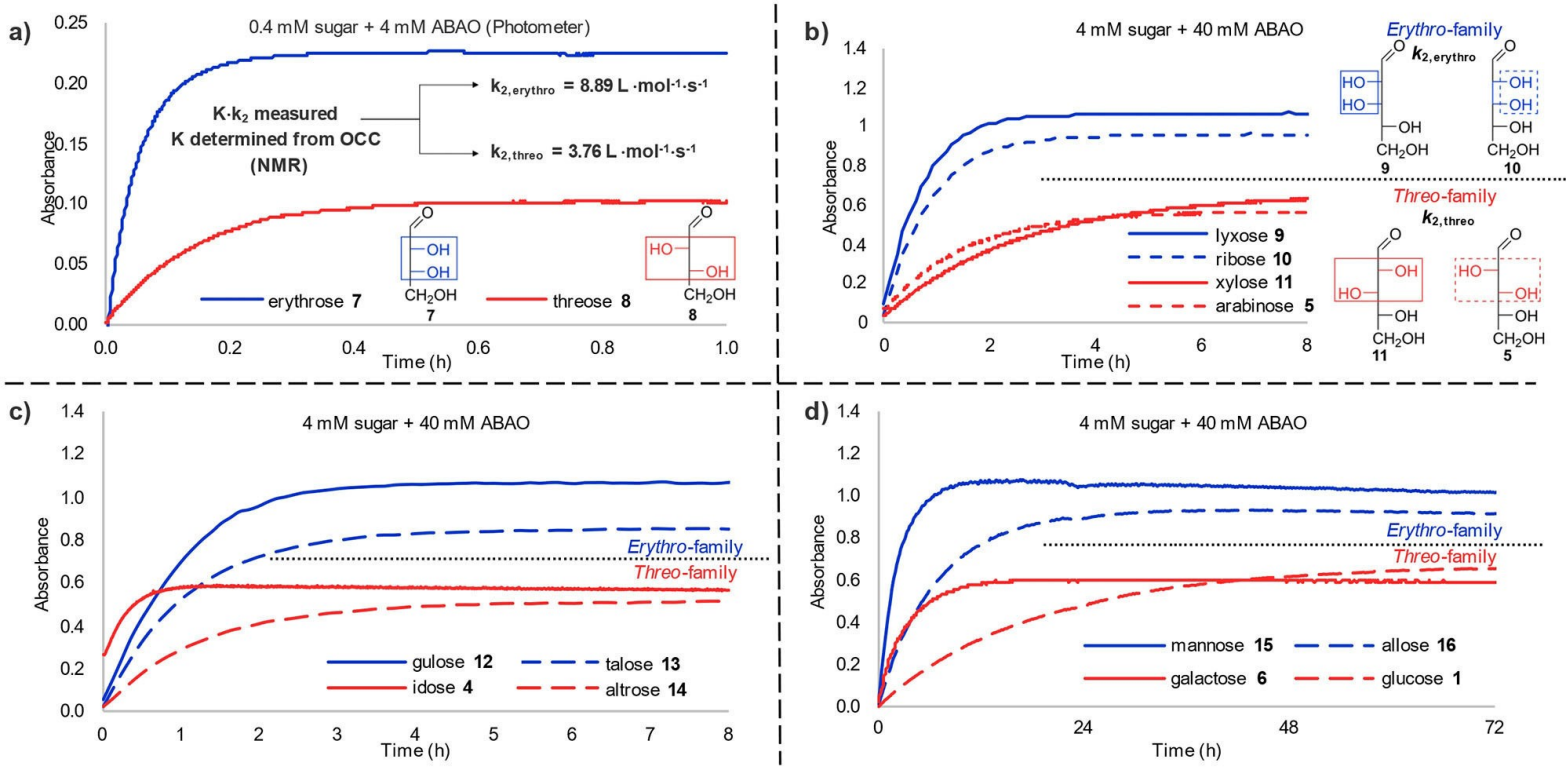
UV‐Curves of ABAO‐adduct formation for all parent aldoses revealing the presence of two families in respect to their relative 2,3‐stereochemistry exhibiting differences in absorption maxima and rate constants. (a) From the tetroses, erythrose **7** and threose **8**, two different rate constants were derived, that were applied for the two families, respectively. (b)–(d) The absorption curves of pentoses and hexoses (fast and slow adduct formation reflecting their OCC values) are depicted exhibiting a clear difference in absorption maxima for the *erythro* and *threo* families again.

In the case of the tetroses, with the reliable OCC values from the ^1^H‐NMR spectra, and the equilibrium constant K can be calculated *via* equation 3, the opposite calculation is possible after transformation to equation 4.

(3) $$\;K=\frac{OCC}{1-OCC}$$

(4) $$\;OCC=\frac{K}{1+K}$$

With known K for the two tetroses, based on the fitting of the curves of formation to deliver α and by using equation 2, k_2_‐values specific for each of the two families were determined (Table 1).

**Table 1:** Stepwise deduction of specific rate konstants k_2_ for the *erythro‐* and *threo‐*families from NMR derived OCC values

Name	OCC [%]	K	α ⋅ 10^3^ [s^−1^]	k_2_ [L ⋅ mol^−1^ ⋅ s^−1^]
l‐erythrose **7**	12.5^[a]^	0.143	5.08	8.89
d‐threose **8**	11.7^[a]^	0.133	2.00	3.76

[a] Determined as points of reference using 1H‐NMR (600 MHz) at 4 mM concentration.

These k_2, erythro_‐, and k_2, threo_‐values (8.89 L ⋅ mol^−1^ ⋅ s^−1^ and 3.76 L ⋅ mol^−1^ ⋅ s^−1^, respectively) were then used for the OCC determination of the other parent aldoses, based on fitting of their curves of formation and utilisation of equation 2 and equation 4.

On a technical note, due to their high OCCs and consequently, very fast conversion at 4 mM, experiments with **7** and **8** were performed at 0.4 mM to generate better analysable data. We confirmed, that the OCC values obtained by the assay are robust to changes in concentration of sugar (0.4 and 4 mM) and ABAO (4 to 40 mM). This was demonstrated for ribose, spanning sugar/ABAO ratios between 1 : 1 and 100 : 1 with only minor changes in OCC (see ESI).

The results of the OCC values of all parent aldoses up to the hexoses are summarised in Table 2 (entry 1–14), clustered by chain length, and sorted by the determined OCC‐values (blue for *erytho*‐ and red for *threo*‐configuration). The obtained numbers were compared to the literature values obtained from the previous NMR studies with 1‐[^13^C]‐labelled monosaccharides^[12]^ displaying a high degree of consistency. The comparison of the absolute values provides good to excellent matches in all cases. Noteworthy, OCCs spanning several orders of magnitude can be obtained with the new method. Further, our assay requires only a few minutes of sample preparation time and relies on a simple photometer compared to the requirement of the synthesis of labelled materials and long measuring times on sophisticated NMR equipment.

**Table 2:** OCC values determined by the ABAO assay and compared to literature values (blue for *erythro*‐ and red for *threo*‐configuration).

Entry	Name	K ⋅ k_2_ ⋅ 10^3^ [L ⋅ mol^−1^ ⋅ s^−1^]	OCC ABAO [%]^[a]^	OCC NMR [%] lit.[^9^, ^12^]
**1**	l‐erythrose **7**	1270	12.5^[b]^	12–12.5
**2**	d‐threose **8**	500	11.7^[b]^	10.6–12
**3**	d‐arabinose **5**	4.78	0.13	0.11
**4**	d‐lyxose **9**	9.78	0.11	0.097
**5**	d‐ribose **10**	8.21	0.092	0.13
**6**	d‐xylose **11**	2.74	0.072	0.071
**7**	l‐idose **4**	26.8	0.71	0.79
**8**	d‐altrose **14**	5.46	0.14	0.093
**9**	l‐gulose **12**	7.77	0.087	0.082
**10**	d‐talose **13**	6.50	0.073	0.081
**11**	d‐galactose **6**	2.04	0.054	0.052
**12**	d‐mannose **15**	2.88	0.032	0.026
**13**	d‐allose **16**	1.03	0.012	0.0095
**14**	d‐glucose **1**	0.37	0.0097	0.010

[a] measured in triplicates with coefficients of variation below 5 % as shown with idose as an example (see ESI). The k_2_‐values used for the determination of the OCCs were selected based on the carbohydrate family with k_2, erythro_=8.89 L ⋅ mol^−1^ ⋅ s^−1^ and k_2, threo_=3.76 L ⋅ mol^−1^ ⋅ s^−1^. [b] Determined as points of reference using 1H‐NMR (600 MHz) at 4 mM concentration (ESI).

With the successful validation of our assay against the reported OCCs of the full standard hexose tree, we set out to apply it to two particular questions from a recent study on the indium‐mediated acyloxyallylation (IMA) of aldoses, which is based on the organometallic addition to the aldehyde moiety. This powerful two‐carbon‐homologation reaction (see Scheme 1 for representative examples) requires a fast consumption of carbohydrate‐based starting material to be successful due to a competing decomposition of the organometallic reagent under the Barbier conditions used.^[3d]^

**Scheme 1:**
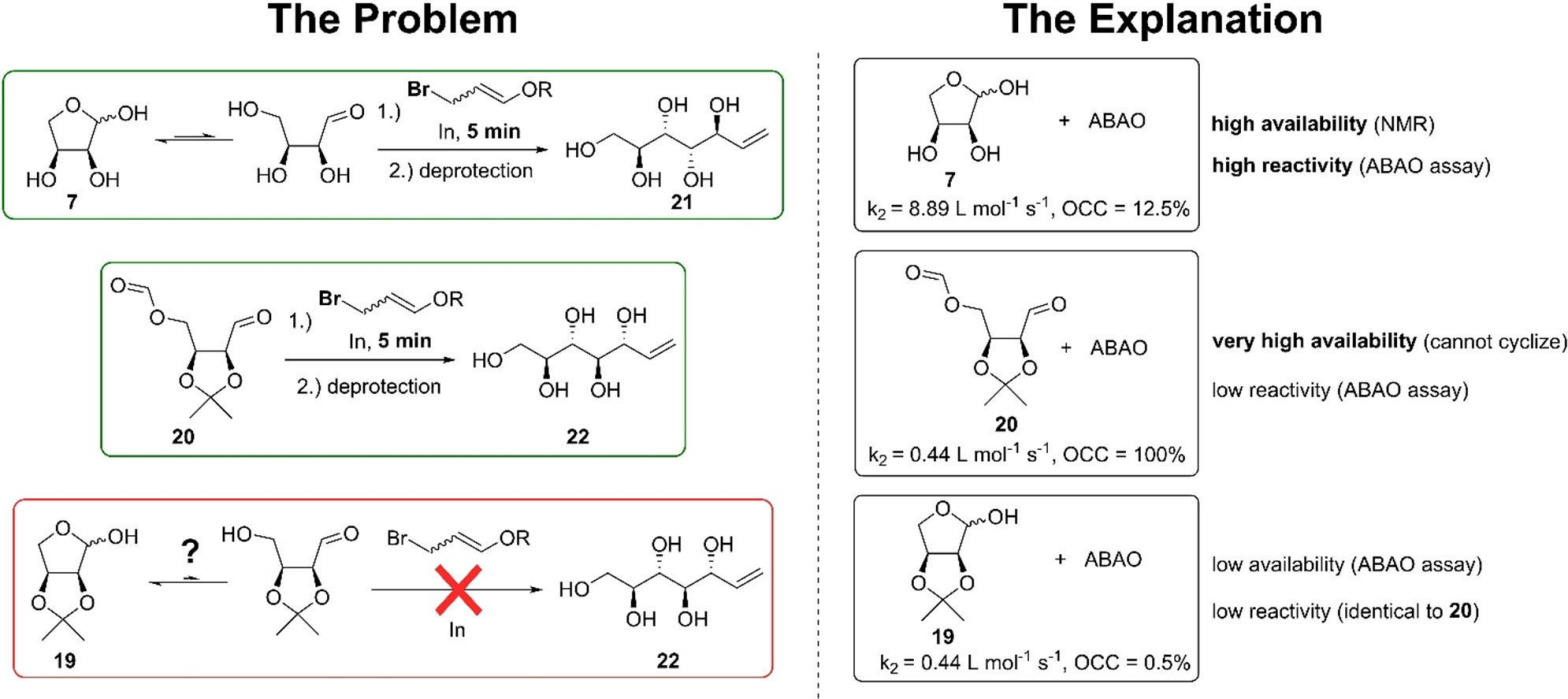
ABAO‐based OCC measurements elucidating the lack of reactivity of reluctant substrate **19** in the indium mediated acyloxyallylation.

This, in our view, represents a prototypic complication when carbohydrates are targeted to react in their (minority) open‐chain form. Nonetheless, we have successfully utilised IMA for the preparation of l‐*glycero*‐d‐*manno*‐heptose **17** at scale amongst more current applications.^[6]^ In such an ongoing study, we required further elongation of heptose **17** as well as its homologous d‐*erythro*‐l‐*manno*‐octose **18** ^[3c]^ (see Figure 4). However, in these IMAs, we observed reduced reactivity compared to their shorter‐chain analogues. We thus used our assay to determine the OCC of heptose **17** and octose **18** in order to gain deeper insights into these observed differences.

**Figure 4:**
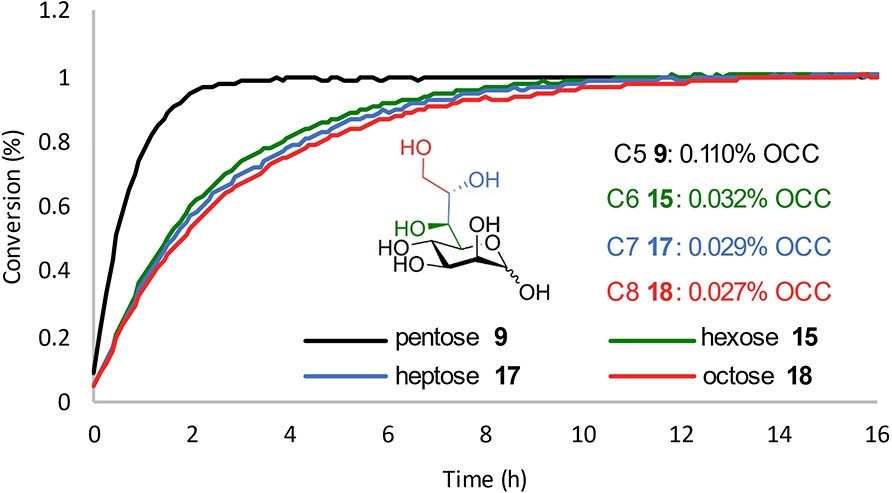
Effect of formal terminal chain elongation onto the OCC of homologous monosaccharides as determined by the ABAO assay.

In turn, we compared their OCCs with the ones of their shorter analogues d‐lyxose **9** and d‐mannose **15** (see Figure 4 and ESI), which share the same relative stereochemistry – (2*O*,3*O*,4*O*)‐*lyxo* in the carbohydrate core. As shown in Figure 4, the pentose lyxose **9** has a roughly 3.5 times higher OCC than its hexose counterpart mannose **15**, which correlates well with their performances in the IMA reaction.^[3c]^ Further formal chain elongation (**17**, **18**) led only to an insignificant change in OCC. Thereby the new assay proved its value to quickly confirm or rule out any effect of the OCC on the observed performance in more complex transformations.

The second application of the ABAO‐assay addressed a more complex question, also related to another aspect of our IMA study. In the systematic investigation of the IMA of reducing sugars, we intensively studied unprotected erythrose **7** as well as 4‐*O*‐formyl‐2,3‐*O*‐isopropylidene erythrose **20**. Upon installation of the protecting group, the diastereodivergent product **22** can be achieved compared to **21** derived from **7**.^[3d]^ However, compound **20** was only ever introduced as a surrogate for its simpler variant 2,3‐*O*‐isopropylidene‐erythrose **19**, which surprisingly and in marked contrast to **7** and **20** could not be converted. Using the new assay, we set out to corroborate our speculations that OCC is responsible with discrete data. Thus, all three compounds of interest were measured with the ABAO‐assay: While **7** and **20** exhibited fast ABAO‐adduct formation, resulting in completion in minutes, **19** was very slowly converted over the course of several hours, even at higher concentration (see time‐courses in the ESI).

Different factors can now be deduced. For erythrose **7**, the combination of a high OCC of 12.5 % and its rate constant in the ABAO‐adduct formation of 8.89 L ⋅ mol^−1^ ⋅ s^−1^ (*vide supra*) confirms the reason for the fast conversion. The 4‐*O*‐formyl‐2,3‐*O*‐isopropylidene‐erythrose **20** showed overall comparable ABAO‐adduct formation. However, it exhibits an OCC of 100 % (as proven by NMR), reflected in a ∼20‐fold lower rate constant of 0.44 L ⋅ mol^−1^ ⋅ s^−1^ (see ESI). This low rate of formation is attributed to the sterically demanding isopropylidene group in close proximity to the carbonyl centre, thus impeding its attack. In analogy to above, we used the rate constant of **20** as a reference for **19** and could thereby deduce the OCC of the protected lactol **19** from its fitted ABAO‐curve. This resulted in a low OCC value of only 0.5 %, thus ∼20 fold lower than in the parent erythrose **7**.

We concluded that as for the case of compound **19**, the combination of the low OCC with a comparably reduced reactivity of the aldehyde moiety is responsible for the observed lack of reactivity, likely due to steric constraints, equally affecting the IMA, (see Scheme 1, bottom right).

In summary, we have shown that the ABAO assay provides a convenient, fast, and reliable approach to determine the minuscule proportions of open‐chain forms of reducing aldoses. It is operationally simple, robust, requires only standard instrumentation, and yielded consistent results to existing methods under substantially reduced effort relying on low‐key equipment. Based on the successful validation against compounds with known OCC‐levels, the determination of first examples of literature unknown OCC‐values was included in this proof‐of‐concept study. More importantly, the second recent synthetic challenge also underlines how the kinetic ABAO‐assay can be used to determine a kind of relative measure of overall aldehyde reactivity. We see great potential in this latter approach as it could be equally applied to other types of aldehydes. Independent of a potential pre‐equilibrium as in aldoses, it could also be used to quantify the effect of steric and electronic effects onto the relative reactivity of a specific aldehyde type.

As for carbohydrates, we are convinced that this new tool will provide a valuable test for chemists to rapidly identify if the OCC is a limitation in their reactions. Thereby, it will help lift a barrier and a bias against using this underutilised compound class in syntheses towards complex organic matter. In this light, our lab is looking deeper into mechanistic details of this powerful interaction, and investigations with a broader scope of carbohydrates and ABAO‐derivatives are also ongoing.

## Conflict of interest

The authors declare no conflict of interest.

## Supporting information

As a service to our authors and readers, this journal provides supporting information supplied by the authors. Such materials are peer reviewed and may be re‐organized for online delivery, but are not copy‐edited or typeset. Technical support issues arising from supporting information (other than missing files) should be addressed to the authors.

Supplementary
