# Supplementary material for: A Kinetic Photometric Assay for the Quantification of the Open‐Chain Content of Aldoses
Source: European J Org Chem. 2021 Apr 8;2021(18):2589–93. doi: 10.1002/ejoc.202001641 (PMC8252792; doi:10.1002/ejoc.202001641)
Supplement: Supplementary file 1 — Supplementary [file EJOC-2021-2589-s001.pdf]

European Journal of Organic Chemistry

Supporting Information

A Kinetic Photometric Assay for the Quantification of the Open-Chain Content of Aldoses

Hubert Kalaus[+], Alexander Reichetseder[+], Verena Scheibelreiter, Florian Rudroff, Christian Stanetty,* and Marko D. Mihovilovic

Supporting Information

Contents

1 General information 3

2 Synthesis and characterization of ABAO 4

3 General procedure for the ABAO assay in the plate reader 5

4 Derivation of the mathematical model describing product absorption 6

5 Determination of OCC shown on the example of ribose 8

5.1 Extraction of the term K·k_2 from the measured absorption spectra 8

5.2 Calculation of OCC values from the term K·k_2 based on the suitable k_2 values 10

5.3 Determination of k2, erythro and k2, threo-values from the measurements of erythrose and threose... 11

5.4 Determination of standard deviations for the case of idose 12

6 All absorption curves, fitted models and OCC calculation 13

6.1 L-Erythrose 7 13

6.2 D-Threose 8.. 13

6.3 D-Lyxose 9... 14

6.4 D-Ribose 10.. 14

6.5 D-Arabinose 5 15

6.6 D-Xylose 11 15

6.7 L-Idose 4 16

6.8 L-Gulose 12.. 16

6.9 D-Talose 13.. 17

6.10 D-Mannose 15 17

6.11 D-Allose 16... 18

6.12 D-Altrose 14 18

6.13 D-Galactose 6 19

6.14 D-Glucose 1.. 20

6.14.1 Mutarotation of α-D-glucopyranose in 100 mM NH_4OAc buffer 20

6.15 L-*Glycero*-D-*manno*-heptose 17 ... 21

6.16 L-*Erythro*-D-*manno*-octose 18 ... 21

6.17 2,3-*O*-Isopropylidene-L-erythrose 19 ... 22

6.18 4-*O*-Formyl-2,3-*O*-isopropylidene-L-erythrose 20 ... 22

7 Comparison regular and normalized data of figure 3 (2O, 3O, 4O-*lyxo*-configured family) 23

8 Influence of ribose and ABAO concentration on rate constant ... 24

9 Comparison between photometer and plate reader ... 24

10 NMR spectra ... 26

10.1 Threose 8 ... 27

10.2 Erythrose 7 ... 28

10.3 ^{1}H-NMR of the ABAO-adduct of erythrose 7 (*erythro*) ... 29

10.4 ^{13}C-NMR of the ABAO-adduct of erythrose 7 (*erythro*) ... 30

10.5 ^{1}H-NMR of the ABAO-adduct of ribose 10 (*erythro*) ... 31

10.6 ^{13}C-NMR of the ABAO-adduct of ribose 10 (*erythro*) ... 32

10.7 ^{1}H-NMR of the ABAO-adduct of mannose 15 (*erythro*) ... 33

10.8 ^{13}C-NMR of the ABAO-adduct of mannose 15 (*erythro*) ... 34

10.9 ^{1}H-NMR of the ABAO-adduct of threose 8 (*threo*) ... 35

10.10 ^{13}C-NMR of the ABAO-adduct of threose 8 (*threo*) ... 36

10.11 ^{1}H-NMR of the ABAO-adduct of arabinose 5 (*threo*) ... 37

10.12 ^{13}C-NMR of the ABAO-adduct of arabinose 5 (*threo*) ... 38

10.13 ^{1}H-NMR of the ABAO-adduct of xylose 11 (*threo*) ... 39

10.14 ^{13}C-NMR of the ABAO-adduct of xylose 11 (*threo*) ... 40

10.15 ^{1}H-NMR of the ABAO-adduct of galactose 6 (*threo*) ... 41

10.16 ^{13}C-NMR of the ABAO-adduct of galactose 6 (*threo*) ... 42

11 Sugars divided into *erythro*- and *threo*-families ... 43

12 References ... 44

1 General information

All chemicals were used directly from commercial sources and used without further purification. NMR spectra were recorded at 297 K in the solvent indicated with an Avance UltraShield 400 and an Avance III HD 600 spectrometer. All spectra were calibrated to the solvent residual peak.[1] Chemical shifts (δ) and coupling constants (J) were expressed in ppm and Hz, respectively. Assignments are based on 2D-NMR (COSY, HSQC, HMBC). Optical rotation was measured on an Anton Paar MCP 500 at the specified conditions, $[\alpha]_D$ values are given in 10^{-1} deg cm^2 g^{-1}. UV/Vis measurements were performed on a platereader Zentyth 3100 from Anthos or on a Shimadzu UV1800 spectrometer equipped with a thermostat at 20 °C. For data analysis the software Graphpad Prism 6 was used.

The compounds L-*glycero*-D-*manno*-heptose **17**,[2] D-*erythro*-L-*manno*-octose **18**,[3] 2,3-*O*-isopropylidene-L-erythrose **19**[4] and 4-*O*-formyl-2,3-*O*-isopropylidene-L-erythrose **20**[4] were prepared according to literature procedures.

2 Synthesis and characterization of ABAO

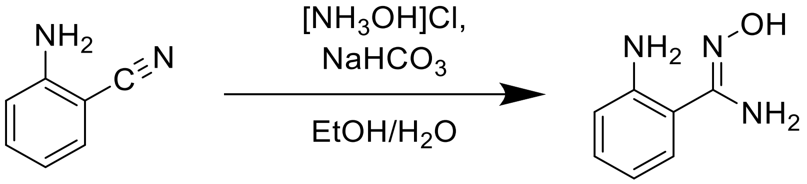

23 **2**

ABAO was prepared according to a modified literature procedure.[5]

The 2-aminobenzonitrile **23** (20.0 g, 169 mmol, 1.00 equiv.) was dissolved in ethanol (330 ml) and hydroxylamine hydrochloride (17.7 g, 254 mmol, 1.50 equiv.), water (100 ml) and $NaHCO_3$ (25.6 g, 305 mml, 1.8 equiv.) were added. The solution was stirred and refluxed for 19 h, when full conversion was observed by TLC (LP:EtOAc 1:1).

The mixture was neutralized with HCl (2 N), monitored using pH paper, and concentrated under reduced pressure. The residue was extracted with EtOAc (5× 75ml), until no further product was detected in the organic phase by TLC (LP:EtOAc 1:1). The combined organic layer was washed with brine, dried over Na_2SO_4 and concentrated. Purification of the crude material was achieved *via* trituration with DCM/LP (1:1, 300 ml, 60 min) yielding 21.8 g (85%) of product **2,** pure according to ^{1}H-NMR.

**Yield**	21.8 g (85%)
**Appearance**	slightly yellow solid
**m.p.**	81.9-84.2 °C (DCM) (Lit.[6] 84-85 °C, benzene)
**TLC**	R_f (LP:EtOAc =1:1) = 0.17
**^{1}H NMR (400 MHz, DMSO-d$_6$)**	δ 5.72 (s, 2H, Ar-N**H**$_2$), 6.21 (s, 2H, C(NOH)N**H**$_2$), 6.53 (td, $J_{H,H}$ = 7.6, 1.3 Hz, 1H, PhH3), 6.66 (dd, J = 8.1, 1.1 Hz, 1H, PhH3), 7.02 (td, J = 7.7, 1.4 Hz, 1H, PhH4), 7.36 (dd, J = 7.9, 1.4 Hz, 1H, PhH6), 9.56 (s, 1H, OH).
**^{13}C NMR (101 MHz, DMSO-d$_6$)**	δ 114.19 (s, PhC1), 114.83 (s, PhC5), 115.43 (s, PhC3), 127.25 (s, PhC6), 128.96 (s, PhC4), 146.76 (s, PhC2), 152.85 (s, **C**(NOH)NH_2).

Spectral data are in accordance with the literature.[7]

3 General procedure for the ABAO assay in the plate reader

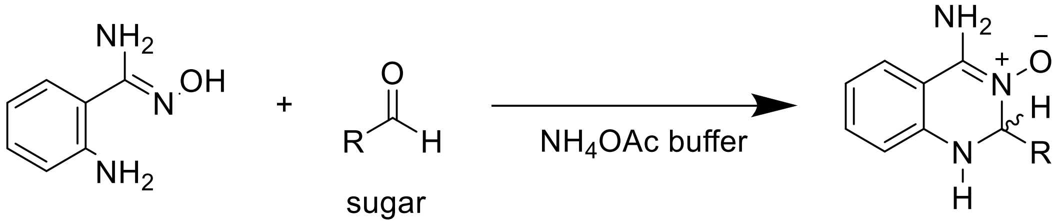

2 **I** **II**

To 190 µl of a solution of 2-aminobenzamidoxime (ABAO, 42.1 mM) dissolved in NH_4OAc buffer (100 mM, pH = 4.5) in a 96-well plate (Greiner, 96-well microplate, PS, F-bottom, clear) with lid (Greiner, with condensation rings, PS, high profile, clear), 10 µl of an aqueous solution of the respective sugar (80 mM) in H_2O was added. While the usage of the lid can be omitted for shorter experiments (<8 h), beyond that point evaporation significantly distorts the obtained curves and the lid significantly improves the result. The plate was shaken in the plate reader and measurements were conducted at 405 nm for the specified time (1 h to 3 days) at 25°C.

Blank samples with addition of pure water instead of the sugar solution were performed and considered. All reactions and the blank samples were performed in triplicates (also see 5.1).

4 Derivation of the mathematical model describing product absorption

The overall reaction between a sugar and ABAO superficially consists of two steps: first, the reversible interconversion between cyclic and the open-chain forms and second the irreversible adduct formation with the ABAO reagent. For a simplified view, the equilibrium is described between all cyclic forms (C; sum of pyranoses and furanoses) and the two open chain forms (O; aldehyde and hydrate) with, k_1 being the rate constant towards the open chain forms, k_{-1} for the respective back-reaction ($K = k_1/k_{-1}$). Further, for the irreversible and rate determining formation of the adduct from the open-chain form (O) and ABAO (A) k_2 is defined with the dehydration of the hydrate to the aldehyde being known to be significantly faster than k_2.[8] The complete deduction of the kinetics of the ABAO-adduct formation step was covered within Kitov's original study with standard aldehydes and is taken as a base and only sketched out herein, but taking into account the pre-equilibrium (of interest).[5]

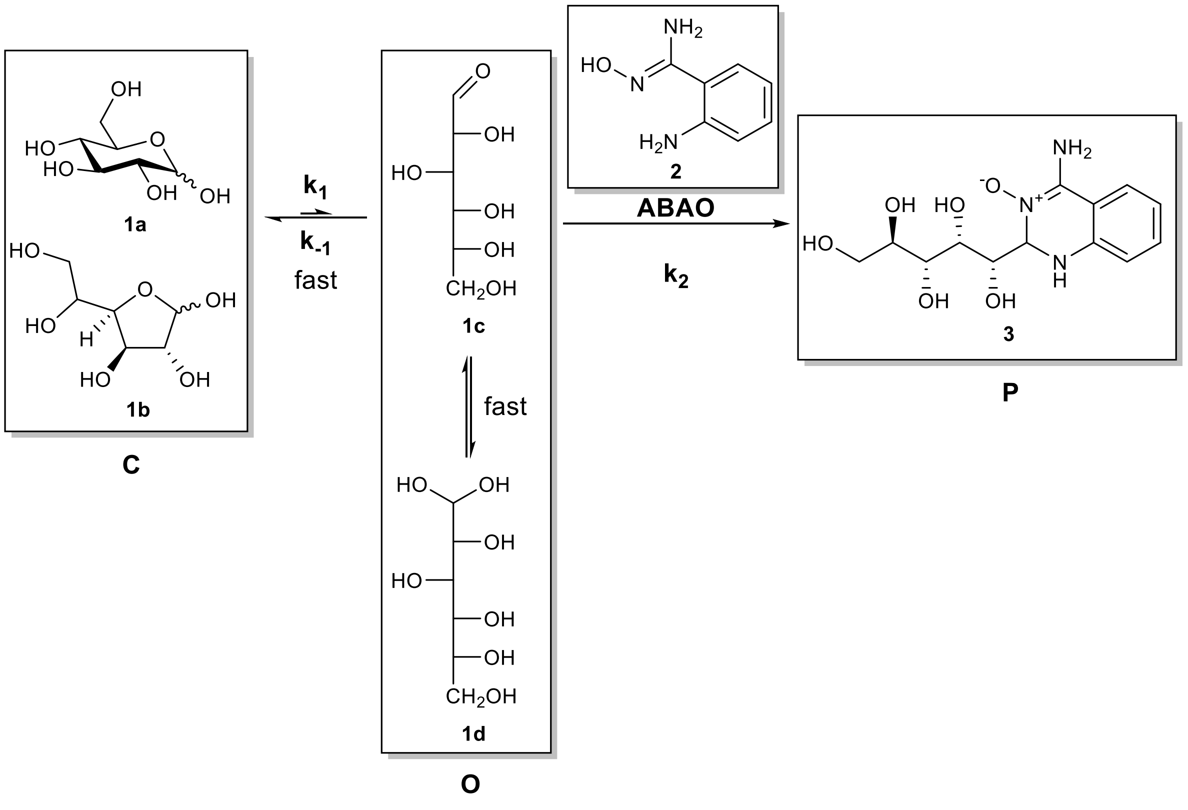

Scheme S1: Depiction of relevant rate constants and introduced abbreviations for the different forms and the product

Or in abbreviated form:

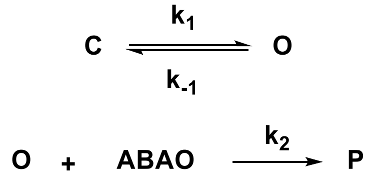

Scheme S2: Essential depiction of elementary reaction steps

In analogy to the case of a classic pre-equilibrium approximation, consequently the following rate law for the formation of the product can be formulated for the ABAO reaction:

Equation S1: $\frac{d[P]}{dt} = k_2 \cdot [O] \cdot [ABAO]$

Under the assumption, that the equilibrium is not affected by the follow-up reaction (which was also confirmed for selected cases *via* NMR-monitoring), the open-chain content can be expressed through the following equation:

Equation S2: $[O] = \frac{k_1}{k_{-1}} \cdot [C] = K \cdot [C]$

This term can be substituted into the product rate equation:

Equation S3: $\frac{d[P]}{dt} = k_2 \cdot K \cdot [ABAO] \cdot [C]$

Next, [C] can be substituted by [C_0]-[P] (neglecting the small open-chain content [O]) to give:

Equation S4: $\frac{d[P]}{dt} = k_2 \cdot K \cdot [ABAO] \cdot ([C]_0 - [P])$

Due to the tenfold excess of ABAO reagent, the ABAO concentration can be assumed as constant over the reaction and the equation can be integrated:

Equation S5: $\frac{d[P]}{[C]_0 - [P]} = k_2 \cdot K \cdot [ABAO] \cdot dt$

Equation S6: $\int \frac{d[P]}{[C]_0 - [P]} = \int k_2 \cdot K \cdot [ABAO] \cdot dt$

Equation S7: $-\ln([C]_0 - [P]) + c = k_2 \cdot K \cdot [ABAO] \cdot t$

Equation S8: $\ln([C]_0 - [P]) = c - k_2 \cdot K \cdot [ABAO] \cdot t$

Equation S9: $[C]_0 - [P] = e^{c - k_2 \cdot K \cdot [ABAO] \cdot t}$

Equation S10: $[P] = [C]_0 - e^c \cdot e^{-k_2 \cdot K \cdot [ABAO] \cdot t}$

With the initial product concentration being zero (P(t = 0) = 0) the specific term for the integration constant c can be determined:

Equation S11: $0 = [C]_0 - e^c \cdot e^{-k_2 \cdot K \cdot [ABAO] \cdot 0}$

Equation S12: $[C]_0 = e^c \cdot e^0 = e^c$

Equation S13: $c = \ln([C]_0)$

And after substituting **Equation S13** into **Equation S10**:

Equation S14: $[P] = [C]_0 - e^{\ln([C]_0)} \cdot e^{-k_2 \cdot K \cdot [ABAO] \cdot t} = [C]_0 - [C]_0 \cdot e^{-k_2 \cdot K \cdot [ABAO] \cdot t}$

Equation S15: $[P] = [C]_0 \cdot \left(1 - e^{-k_2 \cdot K \cdot [ABAO] \cdot t}\right)$

And finally, as at complete conversion $[C]_0 = [P]_{max}$:

Equation S16:$[P] = [P]_{max} \cdot (1 - e^{-k_2 \cdot K \cdot [ABAO] \cdot t})$

With the product being substantially more UV-active at 405 nM absorbtion compared to the starting materials, this equation can be adapted to reflect the measured absorption.

Equation S17: $Abs = Abs_{max} \cdot (1 - e^{-k_2 \cdot K \cdot [ABAO] \cdot t})$

5 Determination of OCC shown on the example of ribose

In the following the determination of the OCC will be shown on one representative example: ribose. All other sugars were processed in accordance.

5.1 Extraction of the term $K \cdot k_2$ from the measured absorption spectra

Below, the measured UV-curve for the ABAO-adduct formation of ribose is depicted, which represents the average of the triplicate measurements after deduction of the equally averaged blank value.

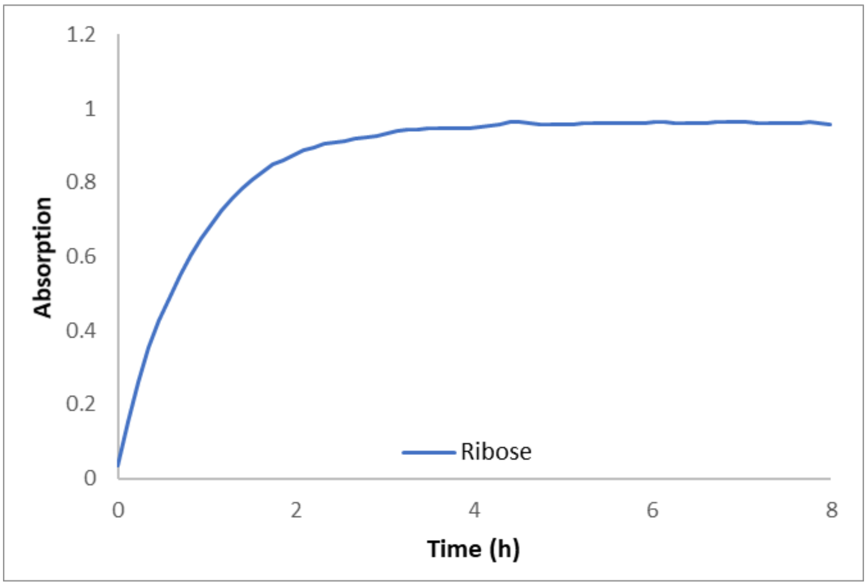

Figure S1: Measured absorption curves of ribose

The datapoints from the measured curve were then fitted using the following general model equation using the program Graphpad Prism 6 (non-linear regression, one-phase association):

$$\textbf{Equation S18: } Y = Y_0 + (Plateau - Y_0) \cdot (1 - e^{-\alpha \cdot x})$$

The obtained values for the Y0, Plateau and α (and Tau = 1/α, half-time = ln(2)/α and Span = Plateau – Y0 as derived values thereof) with their respective standard errors and the coefficient of determination R^2 are given in the following table, on the right side of the graph.

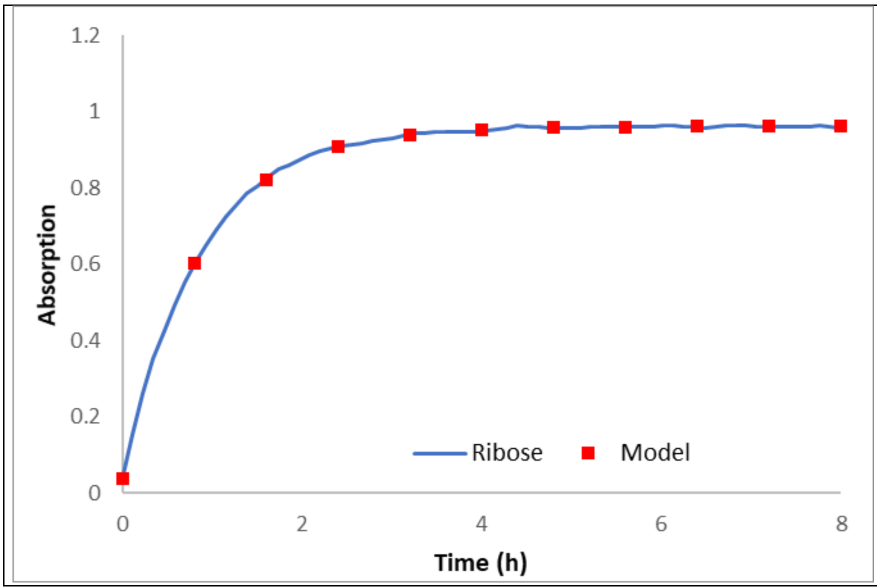

Y0	0.03646
Plateau	0.9613
α	0.0003282
Tau	3047
Half-time	2112
Span	0.9249
**Std. Error**	
Y0	0.001773
Plateau	0.000421
α	0.000001091
Span	0.001747
**Goodness of Fit**	
R square	0.9998

The model's α-value is then equivalent to the term $k_2 \cdot K \cdot [ABAO]$ from **Equation S17**. As the ABAO concentration is known, $k_2 \cdot K$ can be calculated.

Thereby, this mathematical product of values can be determined for each sugar. With the correct k_2 values ($k_{2,\,erythro}$ for the ribose and all threo-configured sugars; $k_{2,\,threo}$ for the threo-configured

sugars) the K value can be calculated which is next translated to the OCC. See 5.3 for the deterimination of $k_{2,\ erythro}$ and $k_{2,\ threo}$.

5.2 Calculation of OCC values from the term K·k_2 based on the suitable k_2 values

Finally, with the two k_2-values at hand, the term k_2·K can be separated. For ribose the $k_{2,\ erythro}$-value of 8.89 L·mol^{-1}·s^{-1} is applicable, as it has a *cis*-2,3-configuration and hence belongs to the *erythro*-family. This gives a K-value of 0.000922.

Transforming the following equation allows to calculate the OCC from the K derived from the model fitting.

Equation S19: $[O] = K \cdot [C]$

Equation S20: $OCC = \frac{[O]}{[O]+[C]}$

Equation S21: $K = \frac{[O]}{[C]} = \frac{OCC}{1-OCC}$

Equation S22: $OCC = \frac{K}{1+K}$

Finally, from this, an OCC-value of 0.0922% for ribose can be calculated using Equation S22.

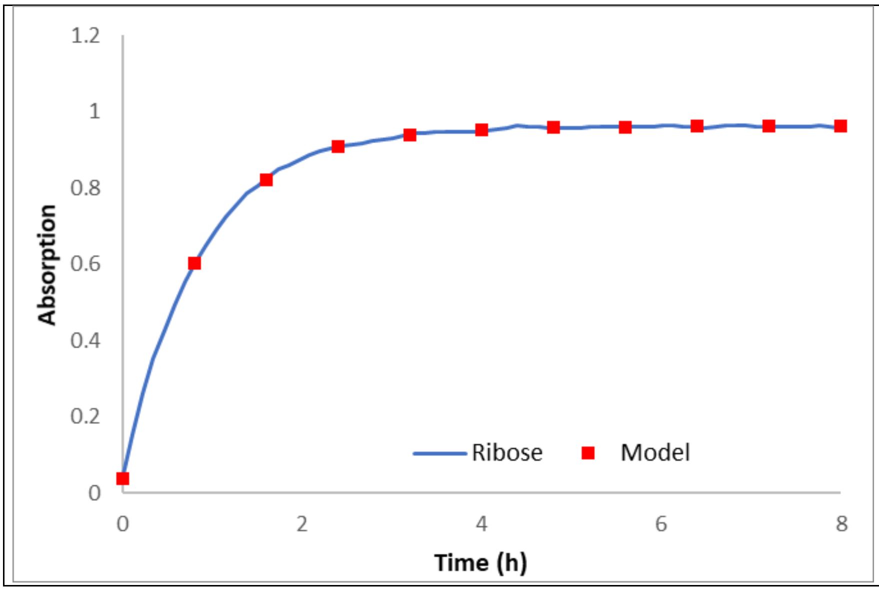

Y0	0.03646
Plateau	0.9613
α	0.0003282
Tau	3047
Half-time	2112
Span	0.9249
**Std. Error**	
Y0	0.001773
Plateau	0.000421
α	0.000001091
Span	0.001747
**Goodness of Fit**	
R square	0.9998
**OCC data**	
K·k_2	0.00821
k_2	8.89
K	0.000922
OCC [%]	0.0922

The numbers for the deduced figures and terms, (K·k_2), k_2; K and OCC are given in the lower right section next to the plotted and modeled absorption for each respective sugar.

5.3 Determination of k2, erythro and k2, threo-values from the measurements of erythrose and threose.

In analogy to the deduction discussed for ribose (section 5), the term $k_2{\cdot}K$ (as described in section 5.1) is determine and in this case, with the (literature) known OCCs (our own NMR experiments, see section 0) for erythrose and threose, the corresponding equilibrium constant K is each deduced (Equation S19-S21). Hence, the following calculations can be made for erythrose: With the measured OCC of 12.5%, a K-value of 0.143 can be calculated with the above equation. Next the respective $k_2{\cdot}K$-value of erythrose can be divided by this K-value to yield the k_2-value of 8.89 $L{\cdot}mol^{-1}{\cdot}s^{-1}$ that was then applied to every sugar *of the erythro*-family.

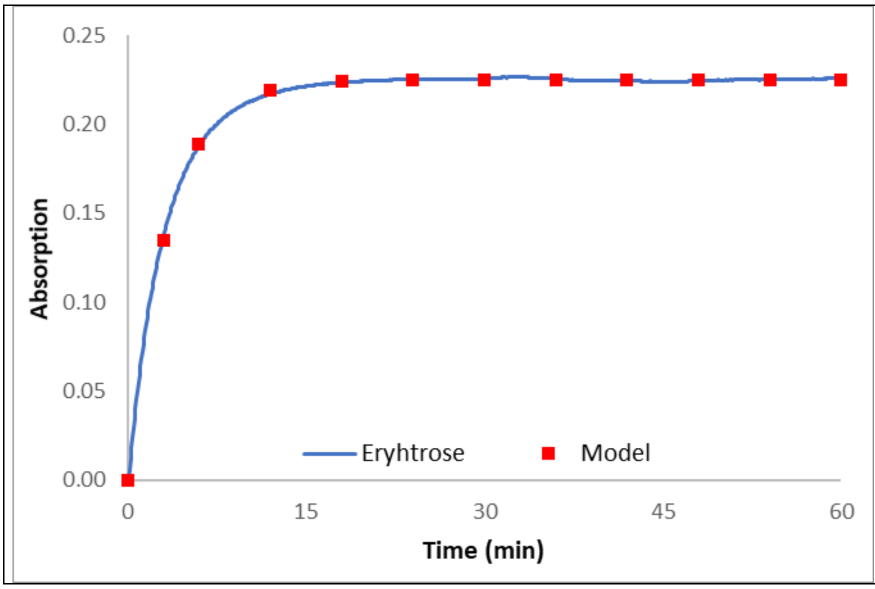

Y0	-0.0003635
Plateau	0.2248
α	0.005079
Tau	196.9
Half-time	136.5
Span	0.2252
**Std. Error**	
Y0	0.0003599
Plateau	0.00004846
α	0.00001231
Span	0.0003569
**Goodness of Fit**	
R square	0.999
**OCC data**	
OCC %	12.5
K	0.143
$K{\cdot}k_2$	1.27
k_2	8.89

Likewise, for threose, the following values were obtained, yielding a $k_{2,\ threo}$ of 3.76 $L{\cdot}mol^{-1}{\cdot}s^{-1}$:

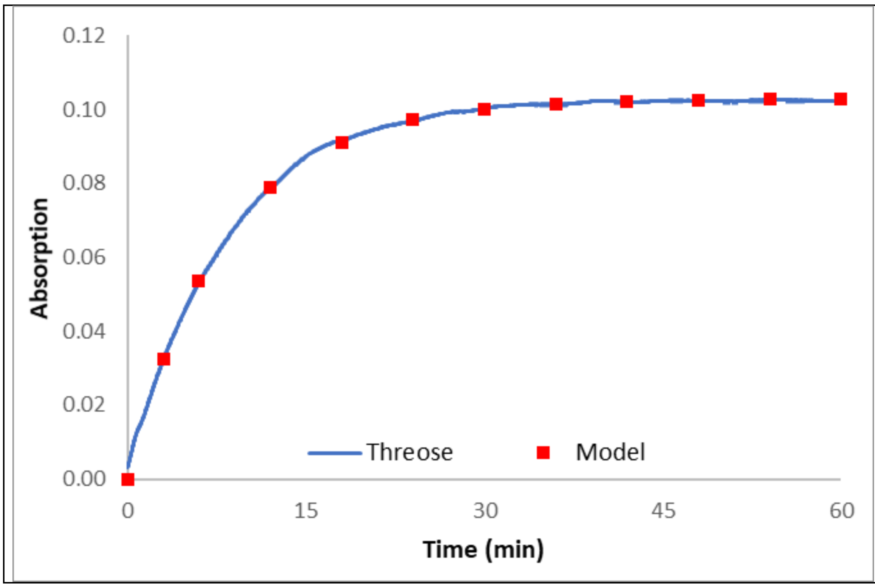

Y0	0.001984
Plateau	0.1028
α	0.001998
Tau	500.5
Half-time	346.9
Span	0.1008
**Std. Error**	
Y0	0.00004205
Plateau	0.0000113
α	0.000001442
Span	0.00004058
**Goodness of Fit**	
R square	0.9996
**OCC data**	
OCC %	11.7
K	0.133
K·k2	0.500
k2	3.76

5.4 Determination of standard deviations for the case of idose

The accuracy of the assay was determined from the triplicates of idose, as this series experiences the greatest inaccuracy. This is due to handling time impacting this measurement the most, as idose is the sugar with the fasted conversion with ABAO investigated herein. For this purpose, each blank was deducted from each of the three curves obtained for idose. The resulting curves are depicted in Figure S2. Following the procedure described in 5.1 and 5.2, an OCC was determined for each of these 9 resulting curves. These OCC values, along with their average, standard deviation and coefficient of variation are depicted in Table S1. The low coefficient of variation, being less than 3%, indicates the high accuracy of the employed method, even for this most effected sugar.

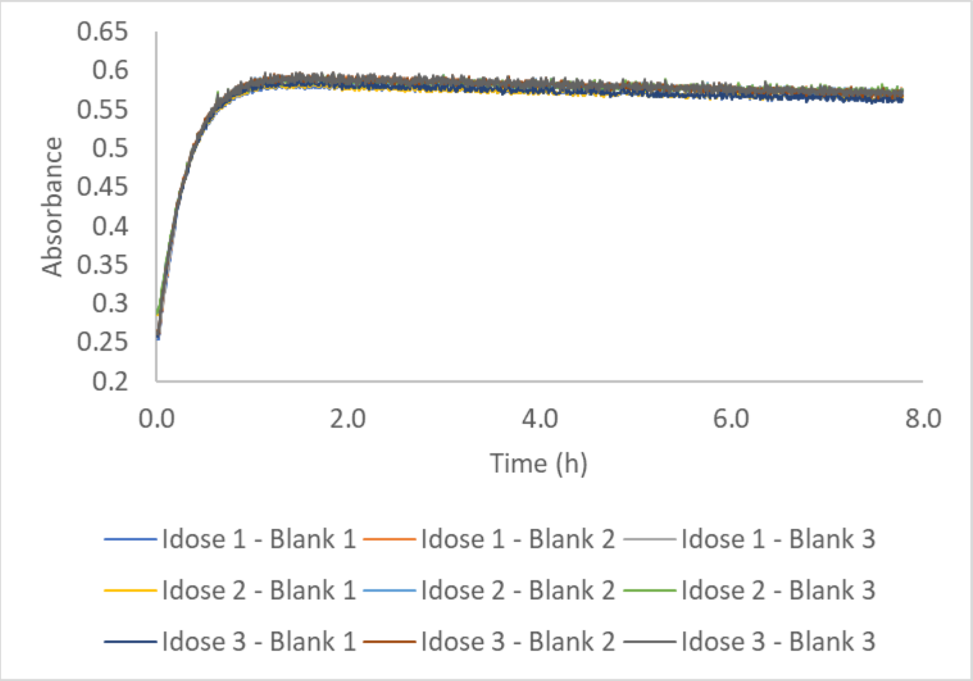

Figure S2: Absorption curves obtained by deducting each of the 3 blanks from each idose measurement.

Table S1: OCC values obtained from the 9 combinations of the 3 idose measurements and 3 blanks, along with their average, standard deviation and coefficient of variation.

OCC (%)	Idose 1	Idose 2	Idose 3
**Blank 1**	0.731	0.694	0.722
**Blank 2**	0.721	0.684	0.712
**Blank 3**	0.709	0.673	0.701
	**Average**	**Standard deviation**	**Coefficient of variation**
	0.705	0.0181	0.0257

6 All absorption curves, fitted models and OCC calculation

6.1 L-Erythrose 7

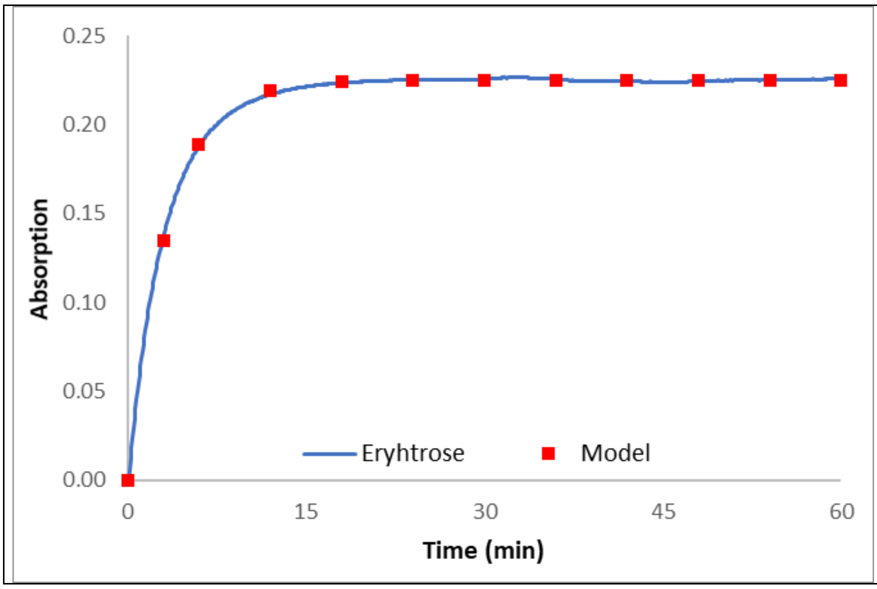

Y0	-0.0003635
Plateau	0.2248
α	0.005079
Tau	196.9
Half-time	136.5
Span	0.2252
**Std. Error**	
Y0	0.0003599
Plateau	0.00004846
α	0.00001231
Span	0.0003569
**Goodness of Fit**	
R square	0.999
**OCC data**	
OCC %	12.5
K	0.143
$K \cdot k_2$	1.27
k_2	8.89

6.2 D-Threose 8

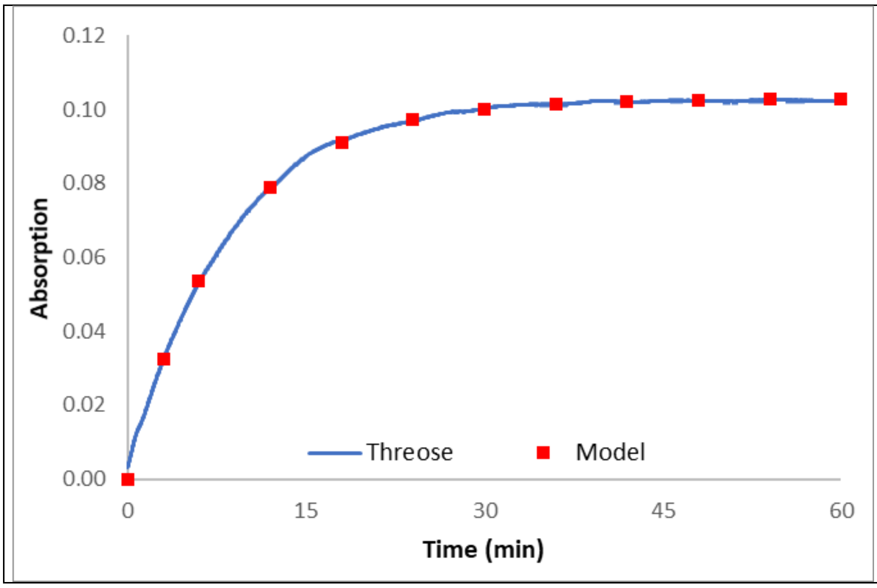

Y0	0.001984
Plateau	0.1028
α	0.001998
Tau	500.5
Half-time	346.9
Span	0.1008
**Std. Error**	
Y0	0.00004205
Plateau	0.0000113
α	0.000001442
Span	0.00004058
**Goodness of Fit**	
R square	0.9996
**OCC data**	
OCC %	11.7
K	0.133
$K \cdot k_2$	0.500
k_2	3.76

6.3 D-Lyxose 9

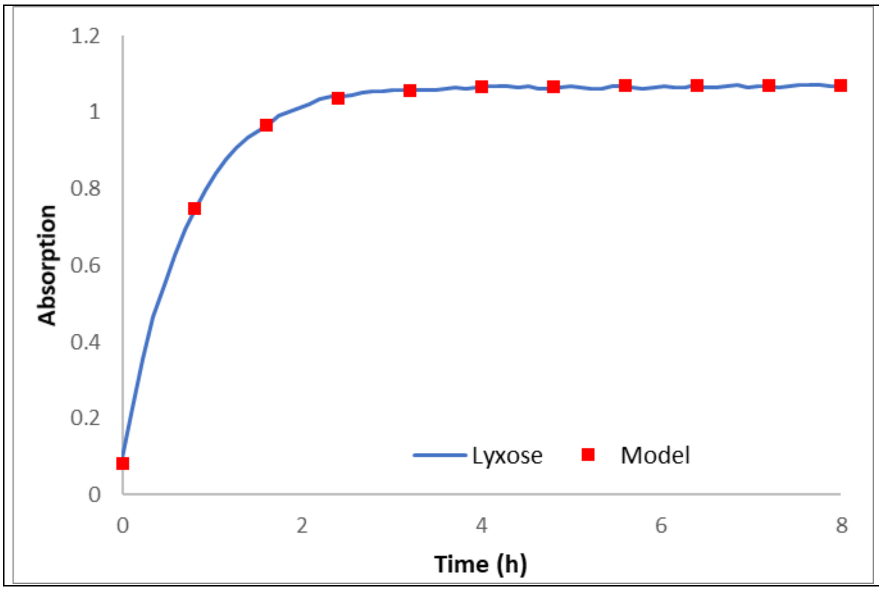

Y0	0.08303
Plateau	1.069
α	0.0003911
Tau	2557
Half-time	1772
Span	0.9855
**Std. Error**	
Y0	0.002115
Plateau	0.0002249
α	1.324E-06
Span	0.00211
**Goodness of Fit**	
R square	0.9994
**OCC data**	
$K \cdot k_2$	0.00978
k_2	8.89
K	0.00110
OCC [%]	0.110

6.4 D-Ribose 10

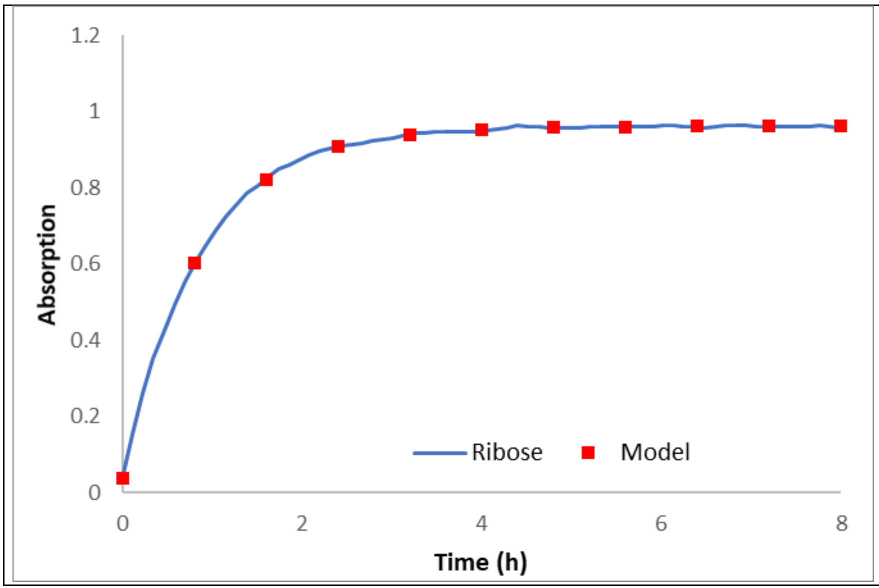

Y0	0.03646
Plateau	0.9613
α	0.0003282
Tau	3047
Half-time	2112
Span	0.9249
**Std. Error**	
Y0	0.001773
Plateau	0.000421
α	0.000001091
Span	0.001747
**Goodness of Fit**	
R square	0.9998
**OCC data**	
$K \cdot k_2$	0.00821
k_2	8.89
K	0.000922
OCC [%]	0.0922

6.5 D-Arabinose 5

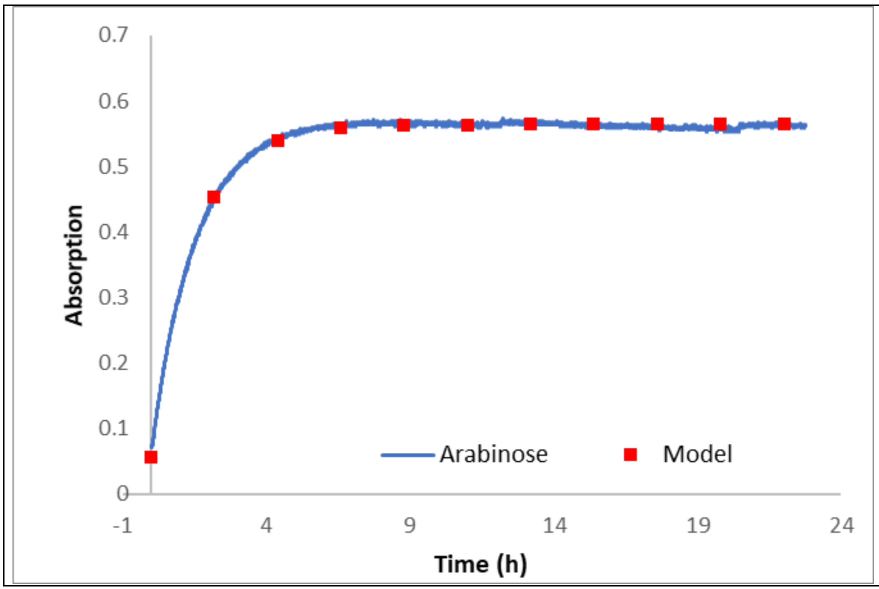

Y0	0.05713
Plateau	0.5642
α	1.91E-04
Tau	5237
Half-time	3630
Span	0.507
**Std. Error**	
Y0	0.0004823
Plateau	0.00007021
α	2.758E-07
Span	0.0004773
**Goodness of Fit**	
R square	0.9982
**OCC data**	
$K \cdot k_2$	0.00478
k_2	3.76
K	0.00127
OCC [%]	0.127

6.6 D-Xylose 11

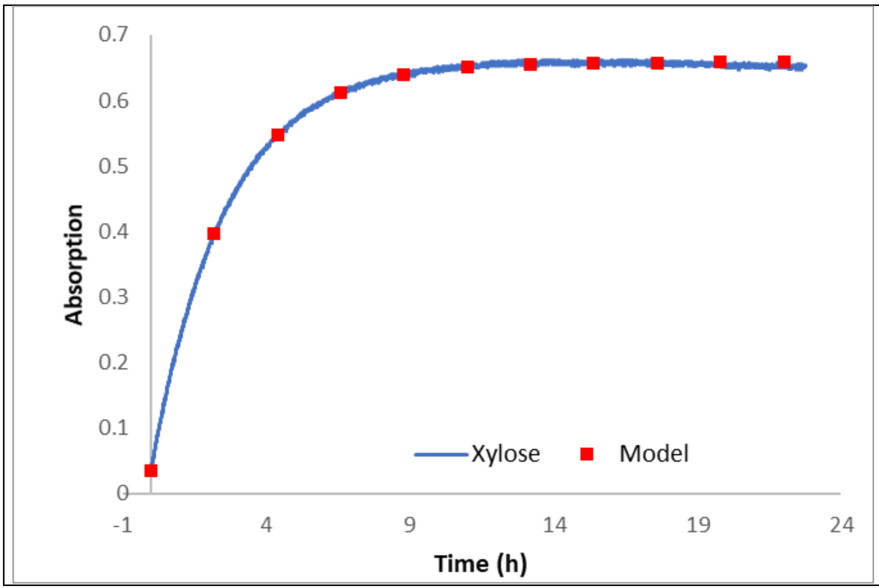

Y0	0.03505
Plateau	0.6582
α	1.09E-04
Tau	9144
Half-time	6338
Span	0.6231
**Std. Error**	
Y0	0.000308
Plateau	0.00006757
α	8.843E-08
Span	0.0003007
**Goodness of Fit**	
R square	0.9995
**OCC data**	
$K \cdot k_2$	0.00274
k_2	3.76
K	0.000725
OCC [%]	0.0725

6.7 L-Idose 4

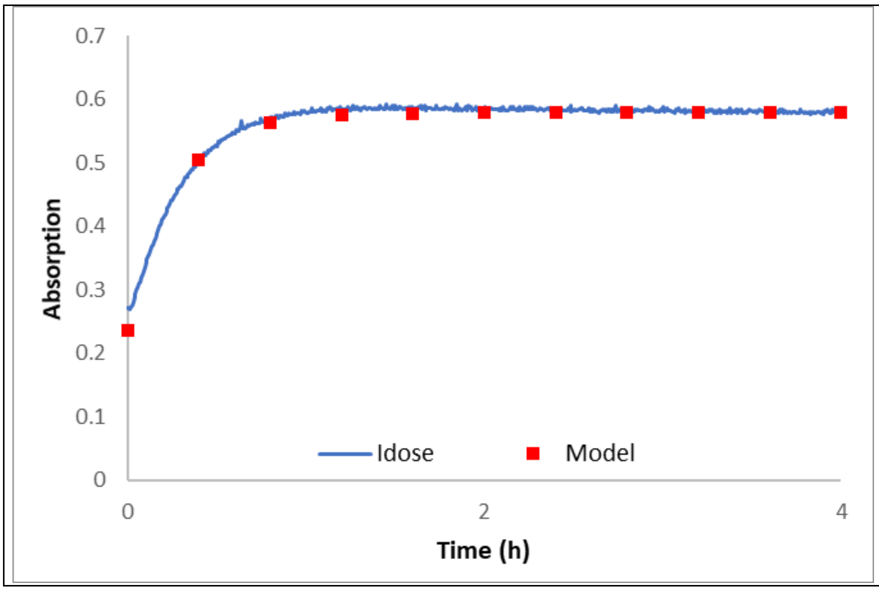

Y0	0.2356
Plateau	0.5788
α	0.001071
Tau	933.9
Half-time	647.3
Span	0.3432
**Std. Error**	
Y0	0.00186
Plateau	0.0001861
α	0.000008584
Span	0.001852
**Goodness of Fit**	
R square	0.981
**OCC data**	
$K \cdot k_2$	0.0268
k_2	3.76
K	0.00705
OCC [%]	0.705

6.8 L-Gulose 12

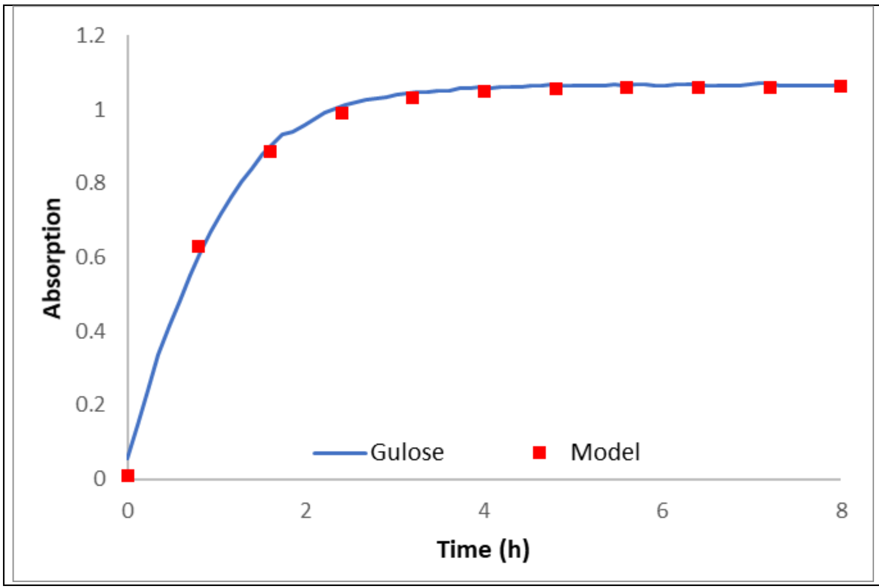

Y0	0.007417
Plateau	1.061
α	0.0003108
Tau	3217
Half-time	2230
Span	1.054
**Std. Error**	
Y0	0.00639
Plateau	0.0008349
α	0.000002974
Span	0.00636
**Goodness of Fit**	
R square	0.9962
**OCC data**	
$K \cdot k_2$	0.00777
k_2	8.89
K	0.000873
OCC [%]	0.0873

6.9 D-Talose 13

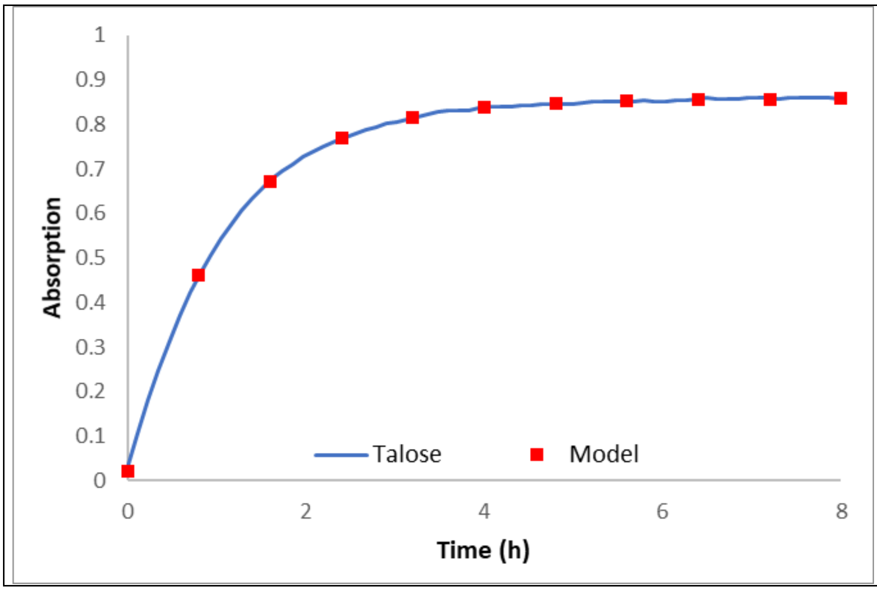

Y0	0.02105
Plateau	0.858
α	2.60E-04
Tau	3844
Half-time	2665
Span	0.837
**Std. Error**	
Y0	0.001356
Plateau	0.0003851
α	7.607E-07
Span	0.001323
**Goodness of Fit**	
R square	0.9999
**OCC data**	
$K \cdot k_2$	0.0065025
k_2	8.89
K	0.000731
OCC [%]	0.0731

6.10 D-Mannose 15

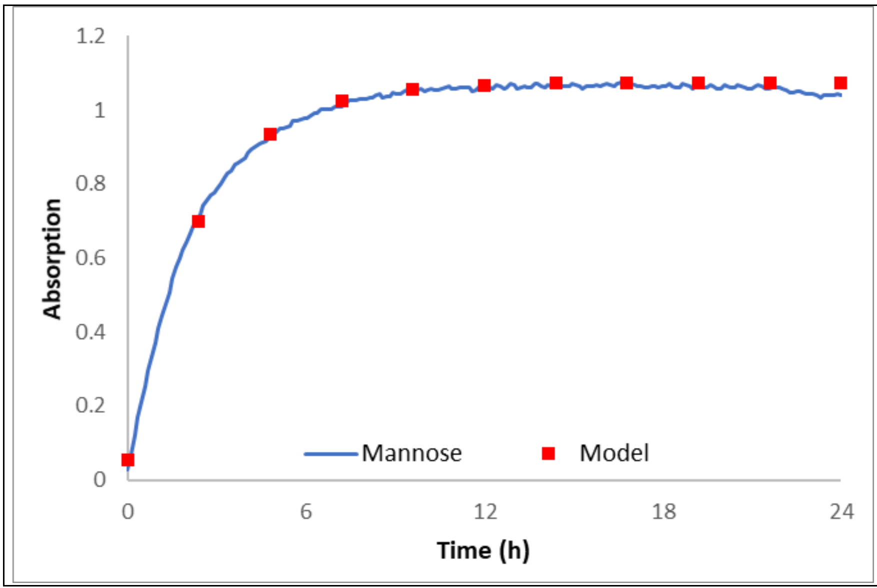

Y0	0.05531
Plateau	1.074
α	0.0001153
Tau	8677
Half-time	6014
Span	1.018
**Std. Error**	
Y0	0.003131
Plateau	0.0009453
α	6.433E-07
Span	0.003021
**Goodness of Fit**	
R square	0.9991
**OCC data**	
$K \cdot k_2$	0.0028825
k_2	8.89
K	0.000324
OCC [%]	0.0324

6.11 D-Allose 16

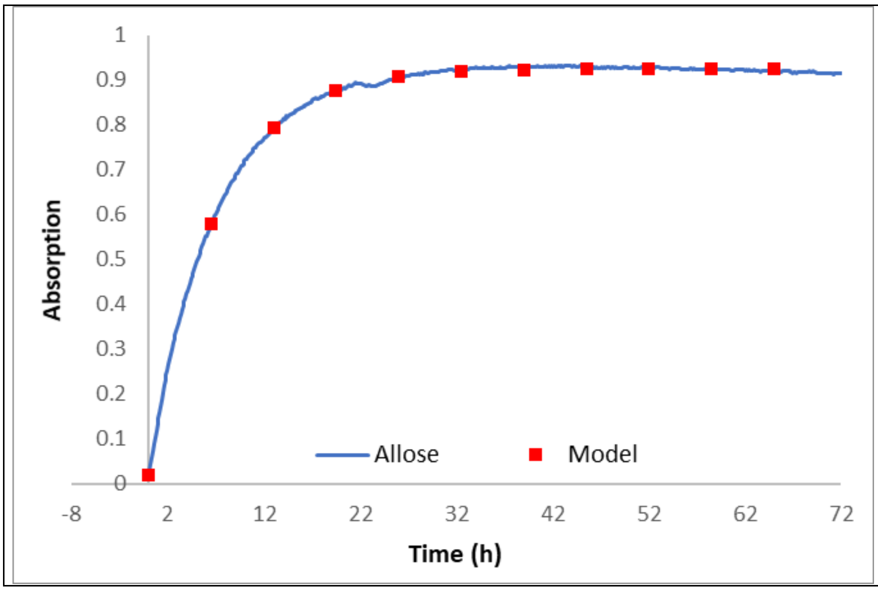

Y0	0.0185
Plateau	0.9263
α	4.11E-05
Tau	24309
Half-time	16850
Span	0.9078
**Std. Error**	
Y0	0.001333
Plateau	0.000255
α	9.623E-08
Span	0.00131
**Goodness of Fit**	
R square	0.9992
**OCC data**	
$K \cdot k_2$	0.0010285
k_2	8.89
K	0.000116
OCC [%]	0.0116

6.12 D-Altrose 14

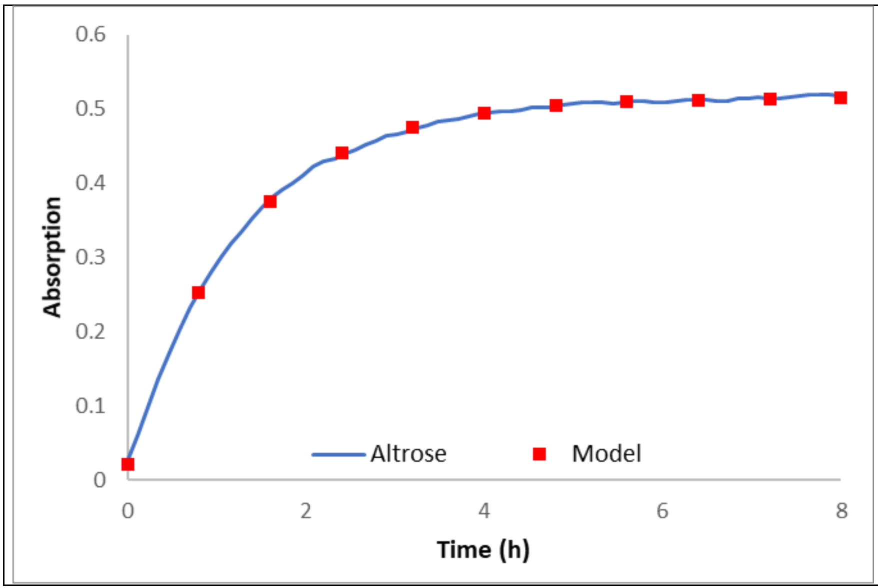

Y0	0.02182
Plateau	0.5158
α	2.18E-04
Tau	4582
Half-time	3176
Span	0.494
**Std. Error**	
Y0	0.001363
Plateau	0.0004564
α	0.000001143
Span	0.001317
**Goodness of Fit**	
R square	0.9996
**OCC data**	
$K \cdot k_2$	0.00546
k_2	3.76
K	0.00145
OCC [%]	0.145

6.13 D-Galactose 6

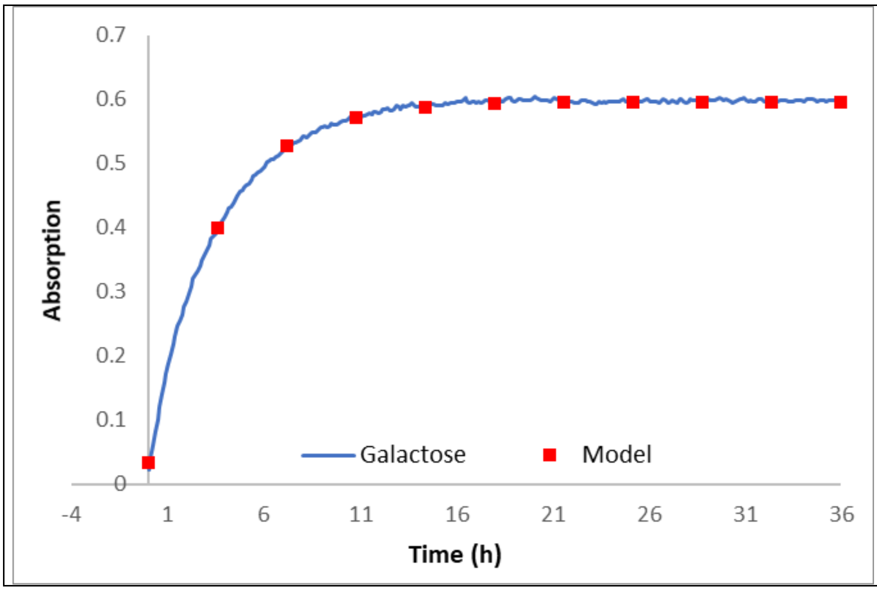

Y0	0.0335
Plateau	0.5962
α	8.15E-05
Tau	12271
Half-time	8506
Span	0.5627
**Std. Error**	
Y0	0.001322
Plateau	0.0001626
α	2.888E-07
Span	0.001313
**Goodness of Fit**	
R square	0.9981
**OCC data**	
$K \cdot k_2$	0.00204
k_2	3.76
K	0.000540
OCC [%]	0.0540

6.14 D-Glucose 1

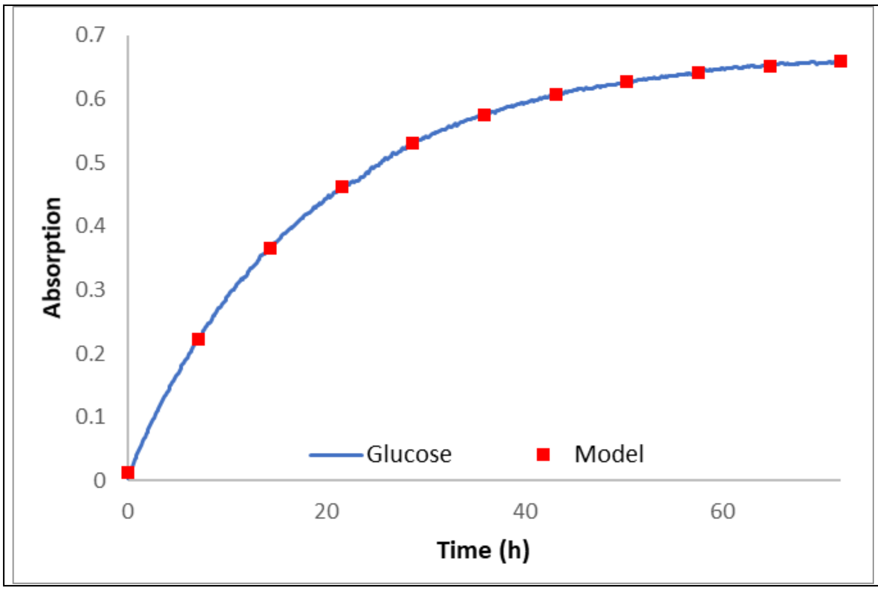

Y0	0.01274
Plateau	0.6736
α	1.47E-05
Tau	68014
Half-time	47144
Span	0.6609
**Std. Error**	
Y0	0.0003675
Plateau	0.0002212
α	1.962E-08
Span	0.0003381
**Goodness of Fit**	
R square	0.9998
**OCC data**	
K·k_2	0.0003675
k_2	3.76
K	0.0000975
OCC [%]	0.00975

6.14.1 Mutarotation of α-D-glucopyranose in 100 mM NH_4OAc buffer

One assumption of the used model described in section 0 was that the adduct formation is significantly slower than the preceding equilibrium step. We chose to support this assumption to be true, even for the slowest case of D-glucose (in the ABAO assay), by measuring the mutarotation and to compare the time it takes for it to complete with the ABAO assay.

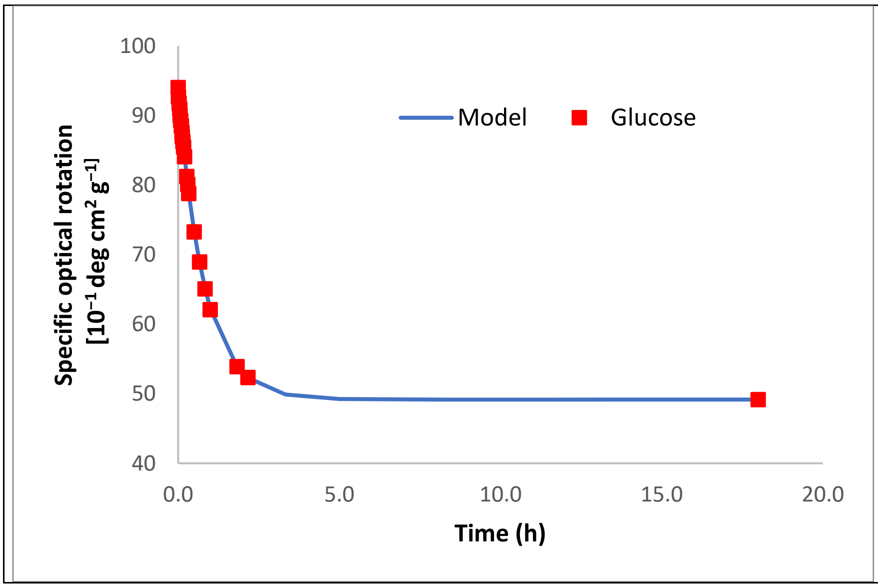

Y0	93.63
Plateau	49.16
α	1.226
Tau	0.816
Half-time	0.5656
Span	-44.47

Therefore, the optical rotation of a freshly prepared α-D-Glucopyranose solution (*c* 1.0) in the employed buffer (100 mM NH_4OAc) was measured over time (*vide infra*). The mutarotation was nearly complete after 2 hours, while the ABAO assay took 72 hours to reach completion,

which strongly corroborates our assumptions. In addition, any superimposed effects based on mutarotation can also be ignored.

6.15 L-*Glycero*-D-*manno*-heptose 17

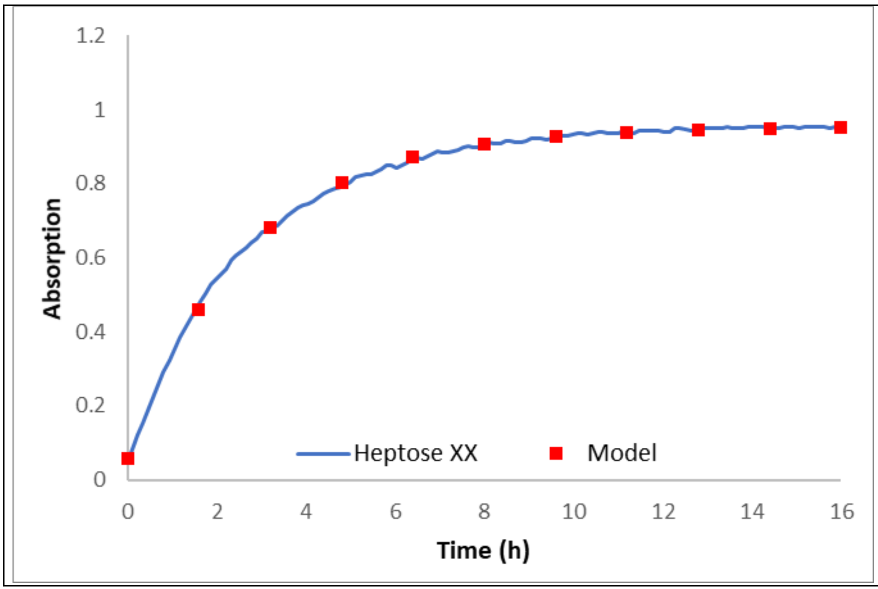

Y0	0.05868
Plateau	0.9524
α	0.0001036
Tau	9656
Half-time	6693
Span	0.8937
**Std. Error**	
Y0	0.002959
Plateau	0.001001
α	6.470e-007
Span	0.002833
**Goodness of Fit**	
R square	0.9989
**OCC data**	
$K\cdot k_2$	0.00259
k_2	8.89
K	0.000291
OCC [%]	0.0291

6.16 L-*Erythro*-D-*manno*-octose 18

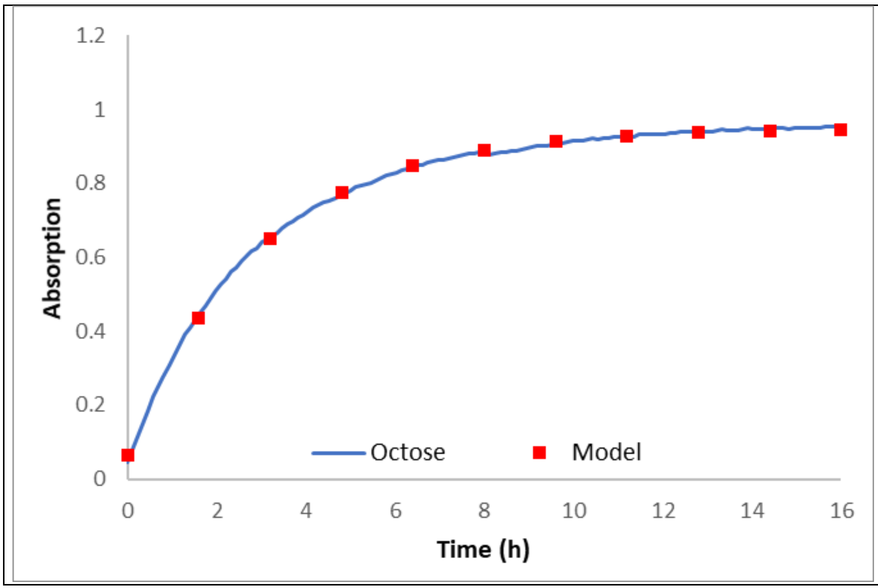

Y0	0.06349
Plateau	0.9472
α	0.00009505
Tau	10521
Half-time	7293
Span	0.8837
**Std. Error**	
Y0	0.003084
Plateau	0.001151
α	6.493E-07
Span	0.002932
**Goodness of Fit**	
R square	0.9988
**OCC data**	
$K\cdot k_2$	0.00238
k_2	8.89
K	0.000267
OCC [%]	0.0267

6.17 2,3-*O*-Isopropylidene-L-erythrose 19

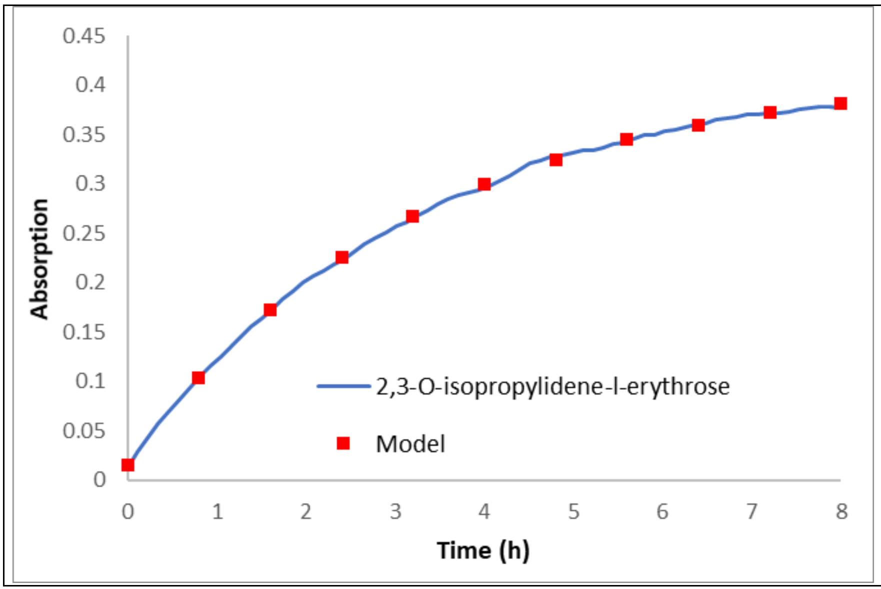

Y0	0.01487
Plateau	0.4144
α	0.00008663
Tau	11544
Half-time	8001
Span	0.3995
**Std. Error**	
Y0	0.0007802
Plateau	0.001043
α	6.258E-07
Span	0.0009253
**Goodness of Fit**	
R square	0.9997
**OCC data**	
$K \cdot k_2$	0.00217
k_2	0.435
K	0.0049
OCC [%]	0.49

6.18 4-*O*-Formyl-2,3-*O*-isopropylidene-L-erythrose 20

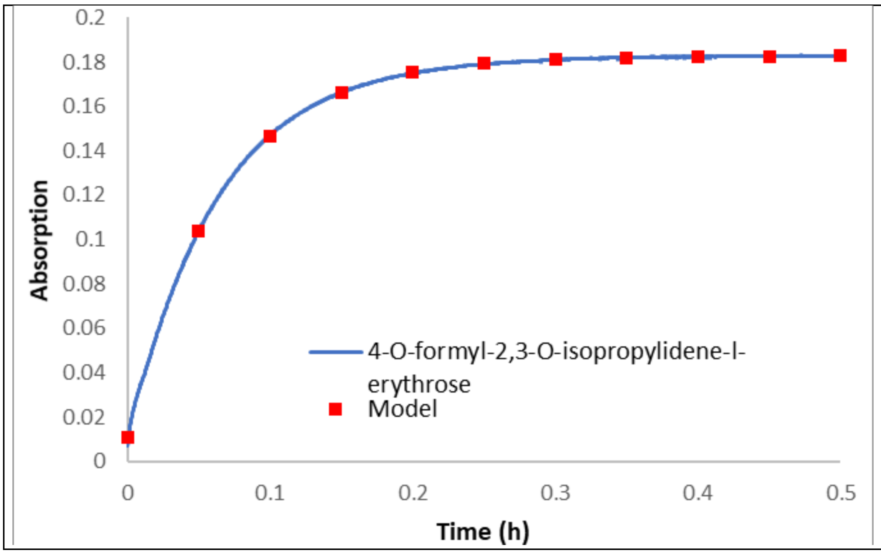

Y0	0.01053
Plateau	0.1827
α	0.004352
Tau	229.8
Half-time	159.3
Span	0.1721
**Std. Error**	
Y0	0.0000477
Plateau	0.00001182
α	2.038E-06
Span	0.00004628
**Goodness of Fit**	
R square	0.9999
**OCC data**	
OCC %	100%
$K \cdot k_2$	0.435
k_2	0.435

7 Comparison regular and normalized data of figure 3 (2O, 3O, 4O-*lyxo*-configured family)

Figure S3 depicts the absorption spectra of the *lyxo*-series in regular form, whereas for the publication a representation with normalized data was chosen (Figure 3 and Figure S4; obtained by scaling each curve to obtain a maximal absorption of 1). Through this visual aid, the relation between the different reaction rates can be more easily graphically deduced, which is why that representation was chosen for the publication. This way it becomes apparent that the k_2 values for **15**, **17** and **18** are very similar but distinctively different form **9**.

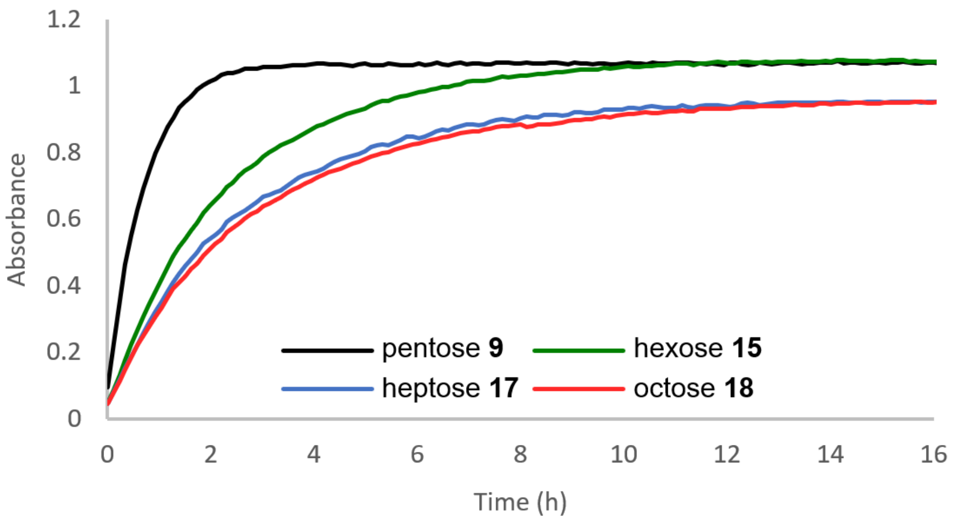

Figure S3: Effect of formal terminal chain elongation onto the OCC of sugars as determined by the ABAO assay without normalization.

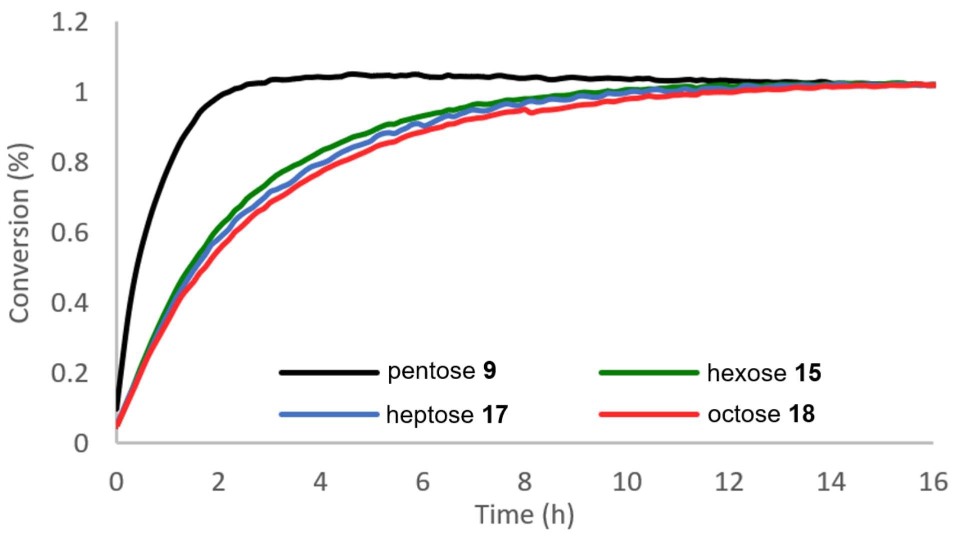

Figure S4: Effect of formal terminal chain elongation onto the OCC of sugars as determined by the ABAO assay after normalization

8 Influence of ribose and ABAO concentration on rate constant

Due to the high reactivity of erythrose and threose, those samples had to be measured at a lower concentration to obtain high quality data. We set out to confirm that the k_2 values determined at this 0.4 mM concentration can be used for the analysis of the curves of the more regular sugars at 4 mM solution a screening. In this light, a screening varying both the concentration of ribose (as a mediocrely fast sugar) and ABAO was conducted. As depicted in the following table, it was found, that the term $K \cdot k_2$ is independent of both the sugar and the ABAO concentration as long as the same ratio of sugar and ABAO was chosen. Presumably, this is due to varying degree of pseudo-first order conditions. Hence, all experiments were undertaken with ratios of 1:10 (sugar : ABAO); 0.4 mM sugar and 4 mM ABAO for erythrose and threose and 4 mM sugar and 40 mM ABAO in all other cases.

$K \cdot k_2$ [$L \cdot mol^{-1} \cdot s^{-1}$]		4	10	20	30	40
Sugar concentration [mM]	4	0.00733	0.00758	0.00746	0.00762	**0.00796**
	3	0.00778	0.00786	0.00794	**0.00801**	0.00793
	2	0.00788	0.00784	**0.00799**	0.00808	0.00861
	1	0.00788	**0.00784**	0.00799	0.00808	0.00919
	0.4	**0.00809**	0.00799	0.00857	0.00948	0.01007

9 Comparison between photometer and plate reader

Generally, the curves measured in the photometer, particularly at lower concentrations are smoother than in the plate reader and there is almost no delay between pipetting and measuring compared to the used plate reader. The recording of multiple samples in the plate reader has a delay, first due to the pipetting of the mixtures and second a delay in the seconds range, caused by the instrument between entering the 96 well plate and the start of the measurement. However, both these effects are only relevant for very fast conversions. On the other hand, in the plate reader up to 32 data points (in triplicates) can be acquired (at much lower amount of sample) compared to only one measurement in the photometer, which are striking benefits of the latter for routing samples. To get an idea about the comparability between plate reader and photometer results, erythrose **7** was measured on both devices at identical concentrations (0.4 mM with 4 mM ABAO). Below, both the regular absorbance (the difference in maximum absorbance is due to differences in layer thickness) and the normalized data (obtained by dividing through the respective maximum value) to show comparability of both data sets which is clearly given.

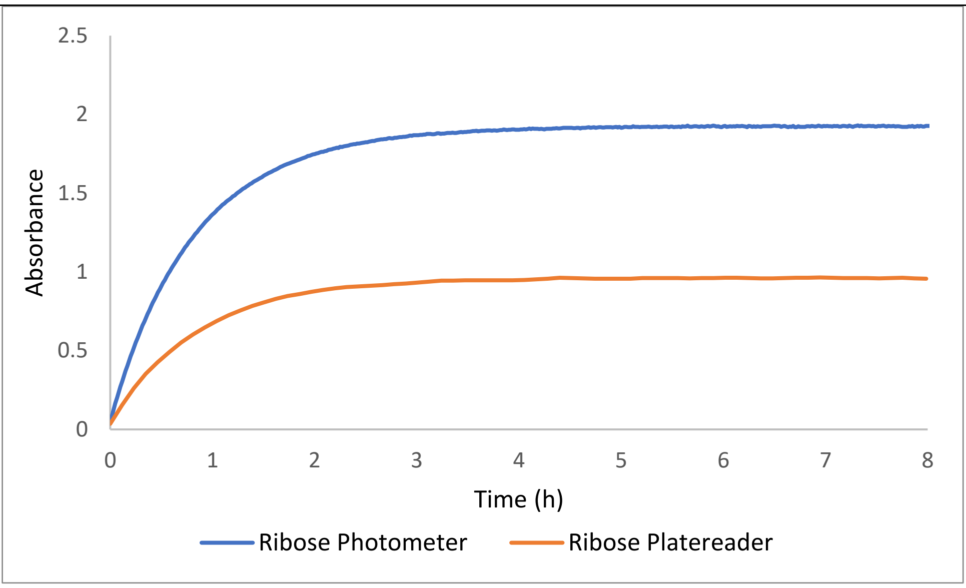

Ribose Photometer		Ribose Photometer	
Y0	0.07233	Y0	0.03646
Plateau	1.923	Plateau	0.9613
**α**	**0.0003306**	**α**	**0.0003282**
Tau	3025	Tau	3047
Half-time	2097	Half-time	2112
Span	1.851	Span	0.9249
**Std. Error**		**Std. Error**	
Y0	0.001074	Y0	0.001773
Plateau	0.0001922	Plateau	0.000421
α	3.023E-07	α	0.000001091
Span	0.001058	Span	0.001747

10 NMR spectra

In this section, two sets of spectra are given:

Firstly, despite the fact that the two tetroses have outstandingly high proportions of open-chain form, the literature values reported for them do vary and are not backed up with a lot of data. Therefore, we decided to determine our own values via ^{1}H-NMR as a base for our assay under conditions most fitting to our study, at 4 mM, which was the concentration under which OCC-values for all other sugars were deduced with the ABAO-assay.

Secondly, the spectra of a representative selection of ABAO-adducts are given. Adducts were prepared from a solution of the respective sugar (300 mM) and ABAO (360 mM) in NH_4OAc buffer (pH 4.5, 100 mM). After 8-72 h, depending on the sugar, excess ABAO was extracted using Et_2O and the aqueous solution was lyophilized. The residue was taken up in D_2O. Further purification was omitted due to the instability of the formed product. Signals of the NMR spectra were labeled in accordance with the following scheme.

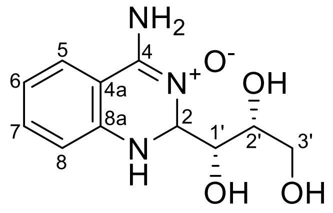

10.1 Threose 8

In order to on the one hand confirm the reported values for threose but also to deduce a most relevant own value for this study, an ^{1}H-NMR spectrum (600 MHz) was recorded at ~4m M concentration. From it, an OCC of 11.7% was determined from the integrals of the respective H1-proton signals (10.7% of the hydrate and 1.0% from the aldehyde form; 38.2% β-furanoside and 50.1% α-furanoside). Due to the vicinity of the dominant water peak and its broad signal, the OCC was also determined and thus confirmed comparing diagnostic peaks more distant to the residual water peak: The H2-protons of both cyclic forms combined give an intensity of 93.0, while the hydrate's H2-proton has an intensity of 11.2 (=10.7%).

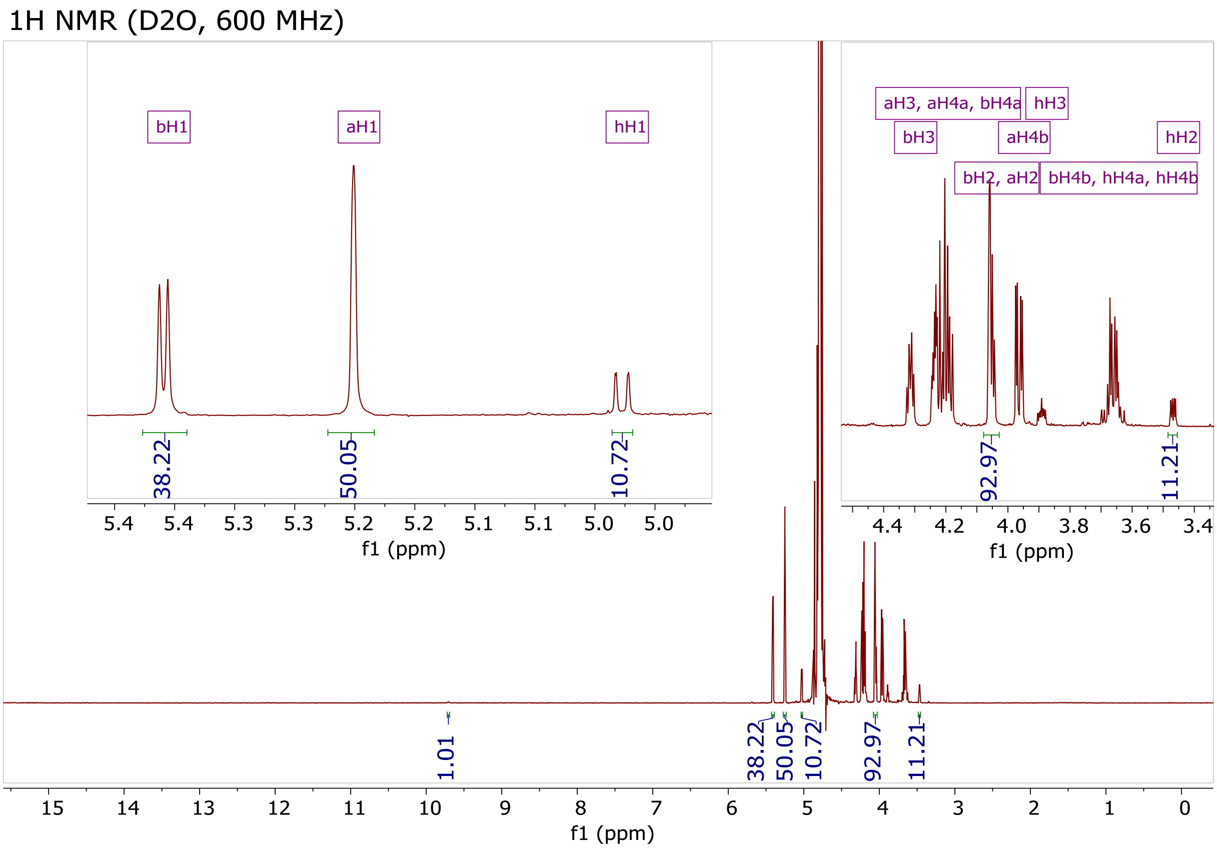

10.2 Erythrose 7

Again, in order to, on the one hand confirm the reported values for erythrose but also to deduce an as most relevant as possible own value for this study, NMR spectra of erythrose in D_2O were measured. As described in the literature, erythrose forms various dimeric and oligomeric structures in more concentrated solution,[9] which is why both the assay and the NMR were measured at low enough concentrations (0.4 mM for the assay, 4 mM for the NMR), where these forms are practically non-existent. Nonetheless, an impurity not ascribed to any monomeric erythrose-form was found in two commercial and one self-prepared sample of erythrose, which had to be considered due to its overlapping signals in the calculation. Doing so, a viable value for the OCC can be determined from the comparison of H1 intensities: 0.23% aldehyde, 12.6% hydrate 25.5% β-furanoside and 61.6% α-furanoside yield an OCC of 12.8%. A second hydrate's signal (H2) with a net-integral of ~12.3 confirms, that the former H1-value is fairly unaffected by the neighboring dominant water signal and we set an OCC of 12.5% as base for our study.

1H NMR (D2O, 600 MHz)

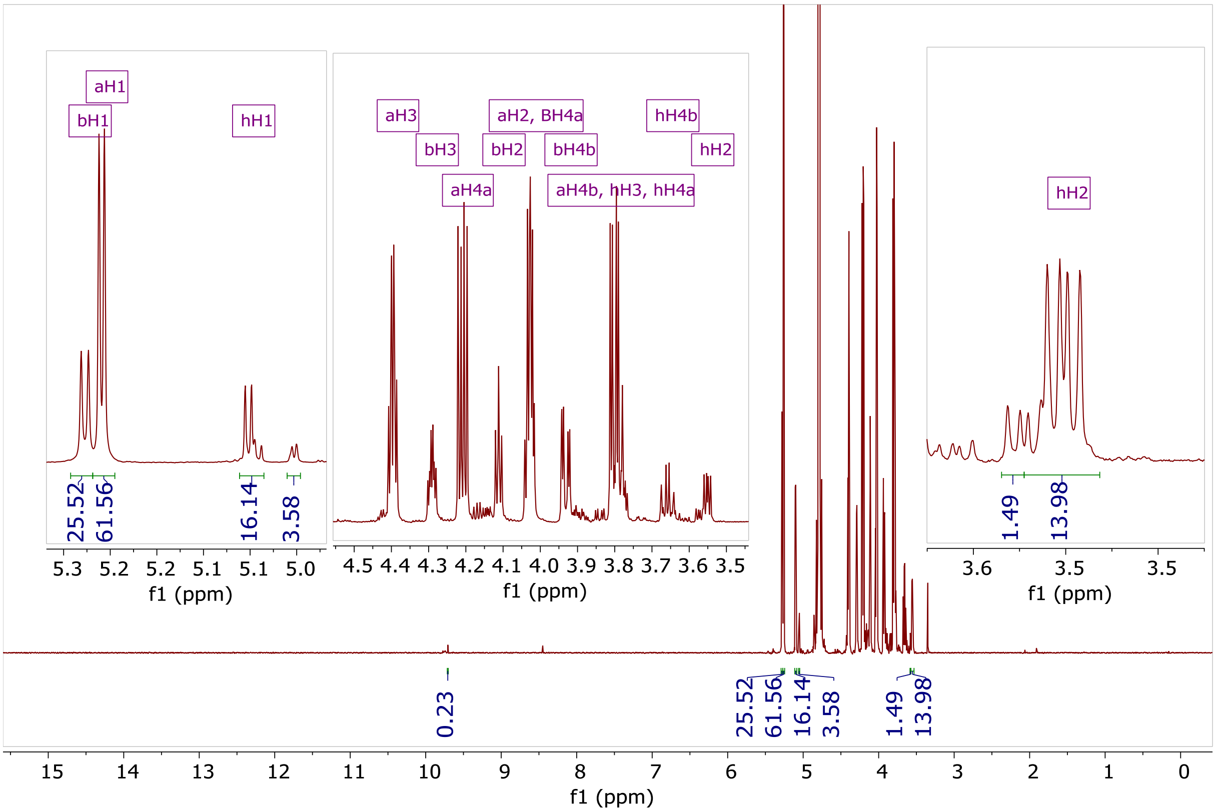

10.3 ^{1}H-NMR of the ABAO-adduct of erythrose 7 (*erythro*)

Epimers were found in a ratio of ~15:85, where epimers' signals are separated, they are assigned as (E1) and (E2). Only the major epimer E2 is annotated:

^{1}H NMR (600 MHz, D_2O) δ 7.33 – 7.26 (m, 1H, H5/H8), 7.24 – 7.16 (m, 1H, H6/H7), 6.83 – 6.66 (m, 2H, H5/H8+H6/H7), 5.19 (d, *J* = 2.2 Hz, 1H, H2(E2)), 3.88 (dd, *J* = 9.3, 2.2 Hz, 1H, H1'(E2)), 3.70 (dd, *J* = 12.0, 2.8 Hz, 1H, H3'a(E2)), 3.67 – 3.61 (m, 1H, H2'(E2)), 3.55 (dd, *J* = 12.0, 5.9 Hz, 1H, H3'b(E2)).

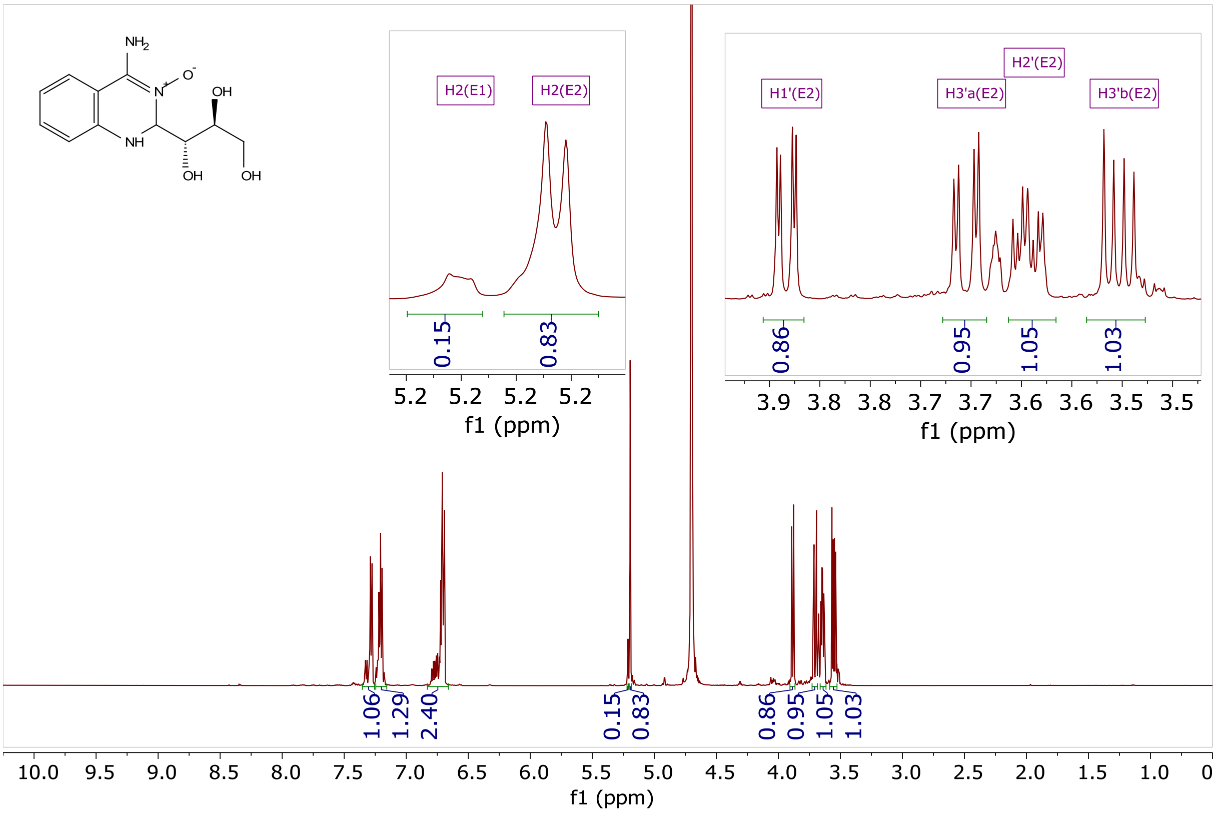

10.4 ^{13}C-NMR of the ABAO-adduct of erythrose 7 (*erythro*)

Only the major epimer E2 was annotated: Epimers were found in a ratio of ~15:85

^{13}C NMR (151 MHz, D_2O) δ 148.57 (C4), 143.08 (C8a), 133.39 (C6/C7), 123.65 (C5/C8), 118.30, 114.52 (C5/C8+C6/C7), 108.30 (C4a), 73.34 (C2), 70.80 (C1'(E2)), 70.01 (C2'(E2)), 62.82 (C3'(E2)).

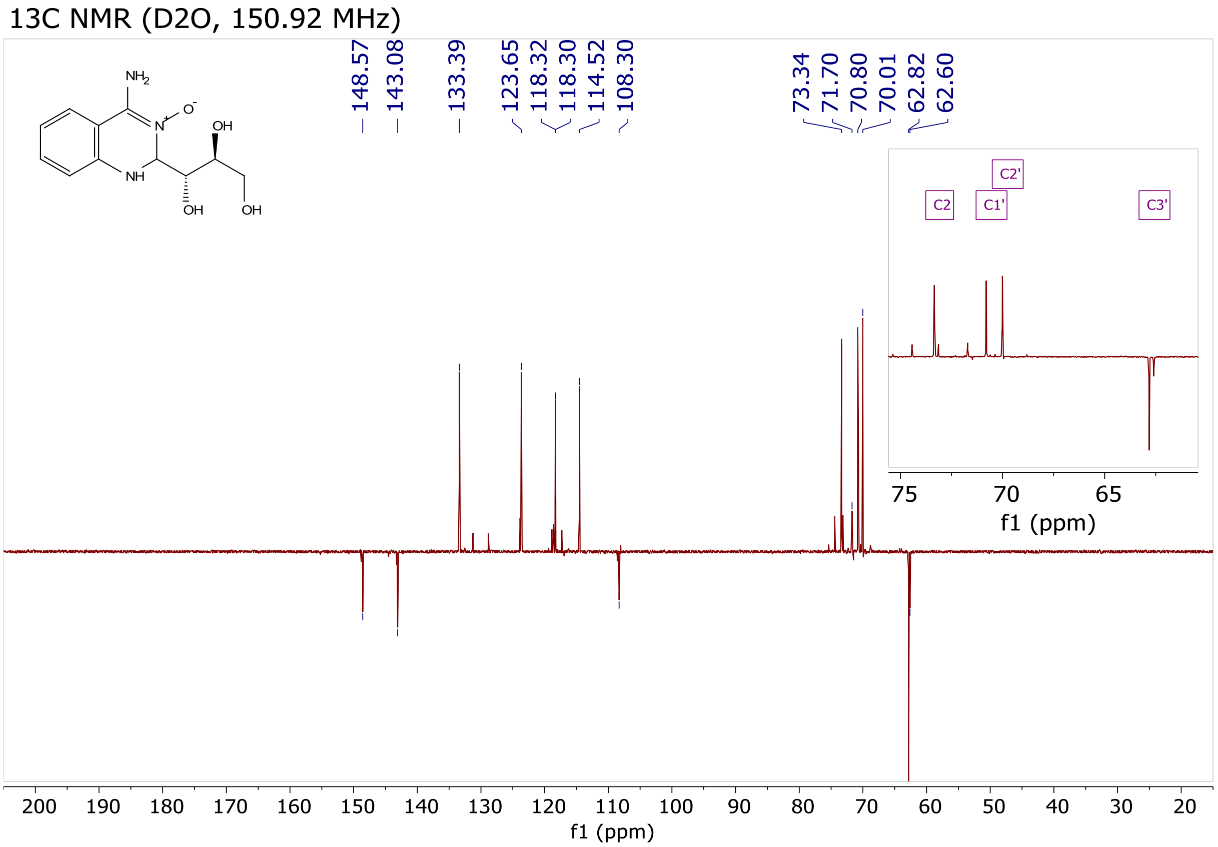

10.5 ^{1}H-NMR of the ABAO-adduct of ribose 10 (*erythro*)

Epimers were found in a ratio of ~20:80, where separated epimers' signals are assigned as (E1) and (E2). Residual acetate from the buffer are still present.

^{1}H NMR (400 MHz, $CDCl_3$) δ 7.49 – 7.41 (m, 1H, H5/H8), 7.36 (m, 1H, H6/H7), 6.92 – 6.81 (m, 2H, H5/H8+H6/H7), 5.36 (d, *J* = 2.6 Hz, 0.2H, H2(E1)), 5.31 (d, *J* = 2.0 Hz, 0.7H, H2(E2)), 4.10 (dd, *J* = 8.7, 2.0 Hz, 0.8H, H1'(E2)), 3.96 (dd, *J* = 6.7, 2.6 Hz, 0.3H, H1'(E1)), 3.90 (m, 1H, H3'), 3.82 (m, 4.3 Hz, 1H, H2'), 3.75 (m, 3.2 Hz, 1H, H4'a), 3.66 (m, 1H, H4'b).

1H NMR (CDCl3, 400.13 MHz)

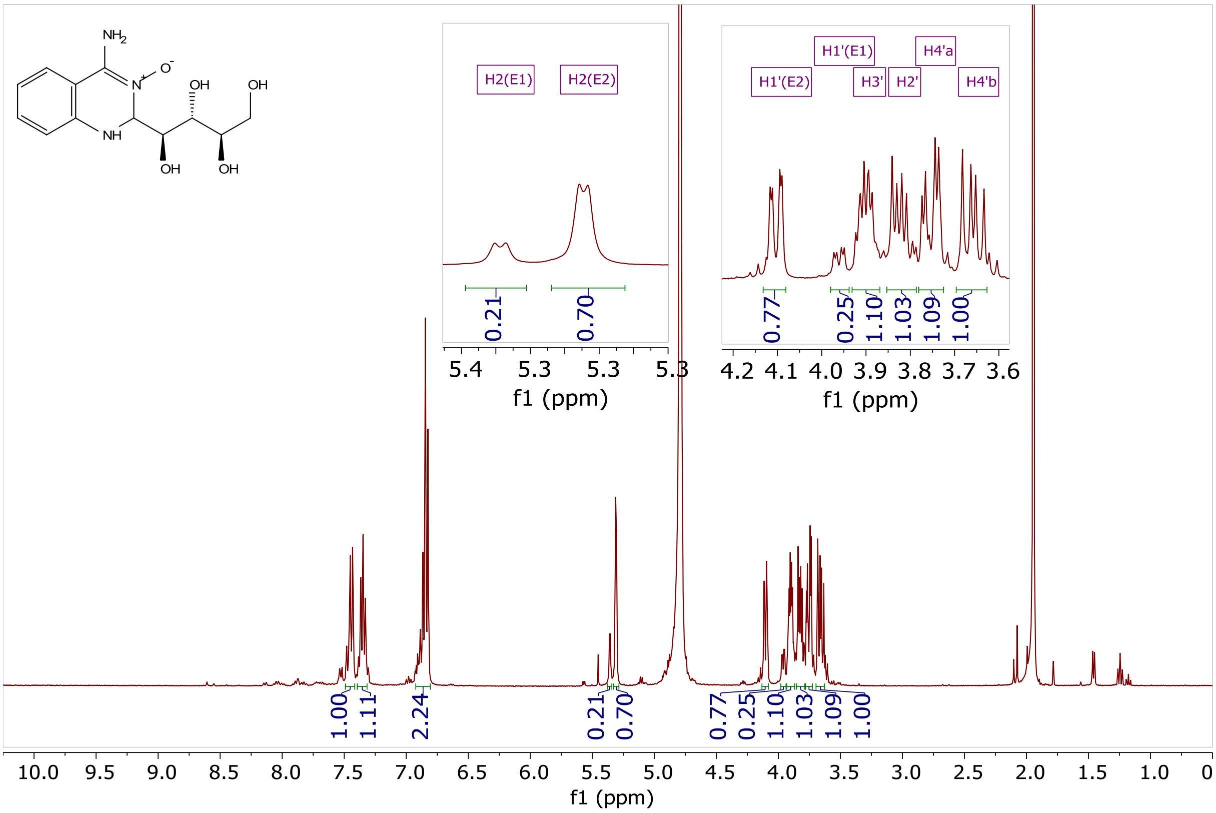

10.6 ^{13}C-NMR of the ABAO-adduct of ribose 10 (*erythro*)

Epimers were found in a ratio of ~20:80, where separated epimers' signals are assigned as (E1) and (E2). Residual acetate from the buffer are still present.

^{13}C NMR (101 MHz, $CDCl_3$) δ 149.7 (C4), 143.5 (C8a), 134.0 (C6/C7(E1)), 133.9 (C6/C7(E2)), 124.2 (C5/C8(E2)), 124.0 (C5/C8(E1)), 118.7 (C5/C6/C7/C8(E1)), 118.6, 114.8 (2x C5/C6/C7/C8(E2)), 114.8 (C5/C6/C7/C8(E1)), 108.1 (C4a), 75.0 (C1'(E1)), 73.6 (C2(E2)), 72.9 (C2(E1)), 72.7 (C3'(E2)), 72.6 (C3'(E1)), 71.7 (C2'(E1)), 71.1 (C1'(E2)), 70.9 (C2'(E2)), 62.6 (C4'(E1)), 61.9 (C4'(E2)).

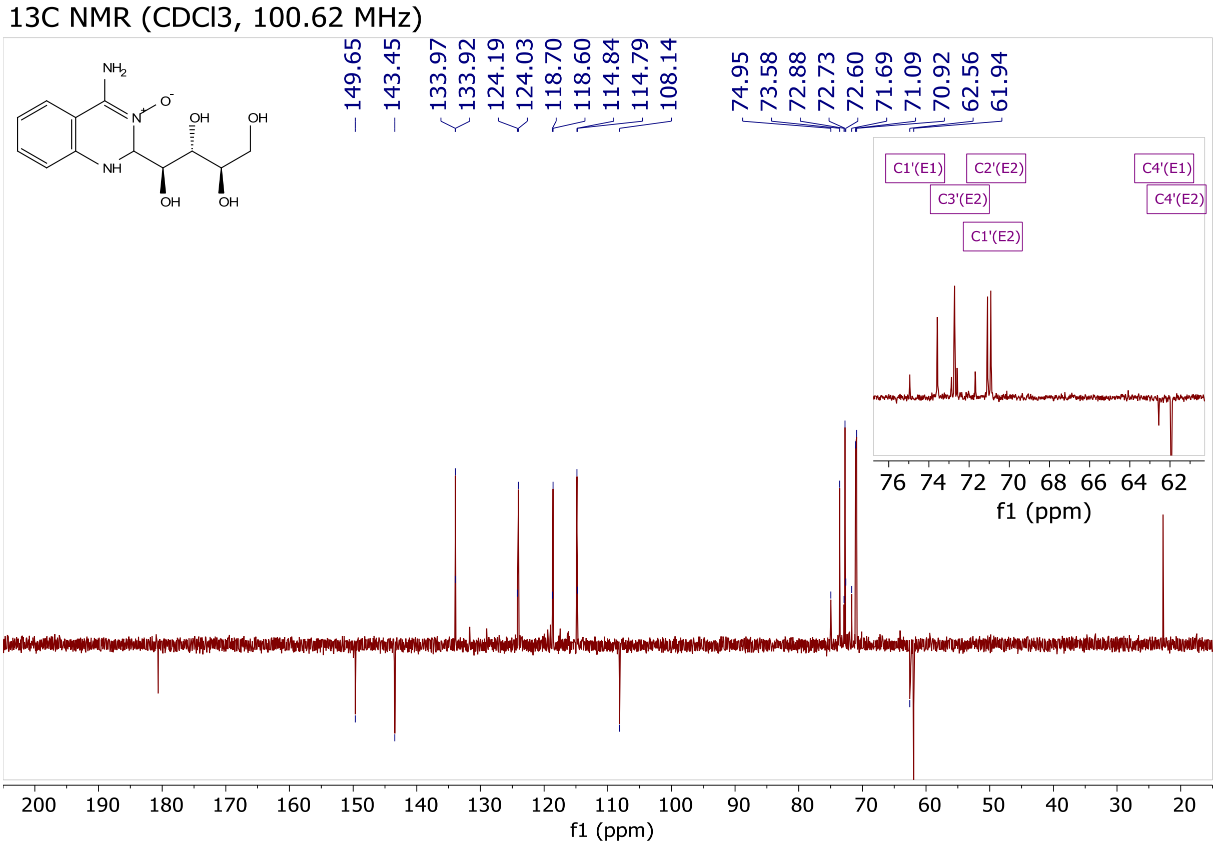

10.7 ^{1}H-NMR of the ABAO-adduct of mannose 15 (*erythro*)

Epimers were found in a ratio of ~15:85, where separated epimers' signals are assigned as (E1) and (E2). Residual amounts of acetate, mannose, and a decomposition product are present.

^{1}H NMR (600 MHz, D_2O) δ 7.45 (m, 1H, H5/H8), 7.35 (m, 1H, H6/H7), 6.85 – 6.76 (m, 2H, H5/H8+H6/H7), 5.49 (d, *J* = 2.2 Hz, 0.2H, H2(E1)), 5.41 (d, *J* = 1.7 Hz, 0.8H, H2(E2)), 4.14 (dd, *J* = 9.9, 1.7 Hz, 0.8H, H1'(E2)), 4.03 (dd, *J* = 9.6, 0.9 Hz, 0.2H, H1'(E1)), 3.95 (d, *J* = 10.0 Hz, 1H, H2'), 3.85 (dd, *J* = 11.8, 2.5 Hz, 1H, H5'a), 3.76 – 3.73 (m, 2H, H3'+H4'), 3.66 (dd, *J* = 11.9, 5.5 Hz, 1H, H5'b).

1H NMR (D2O, 600.15 MHz)

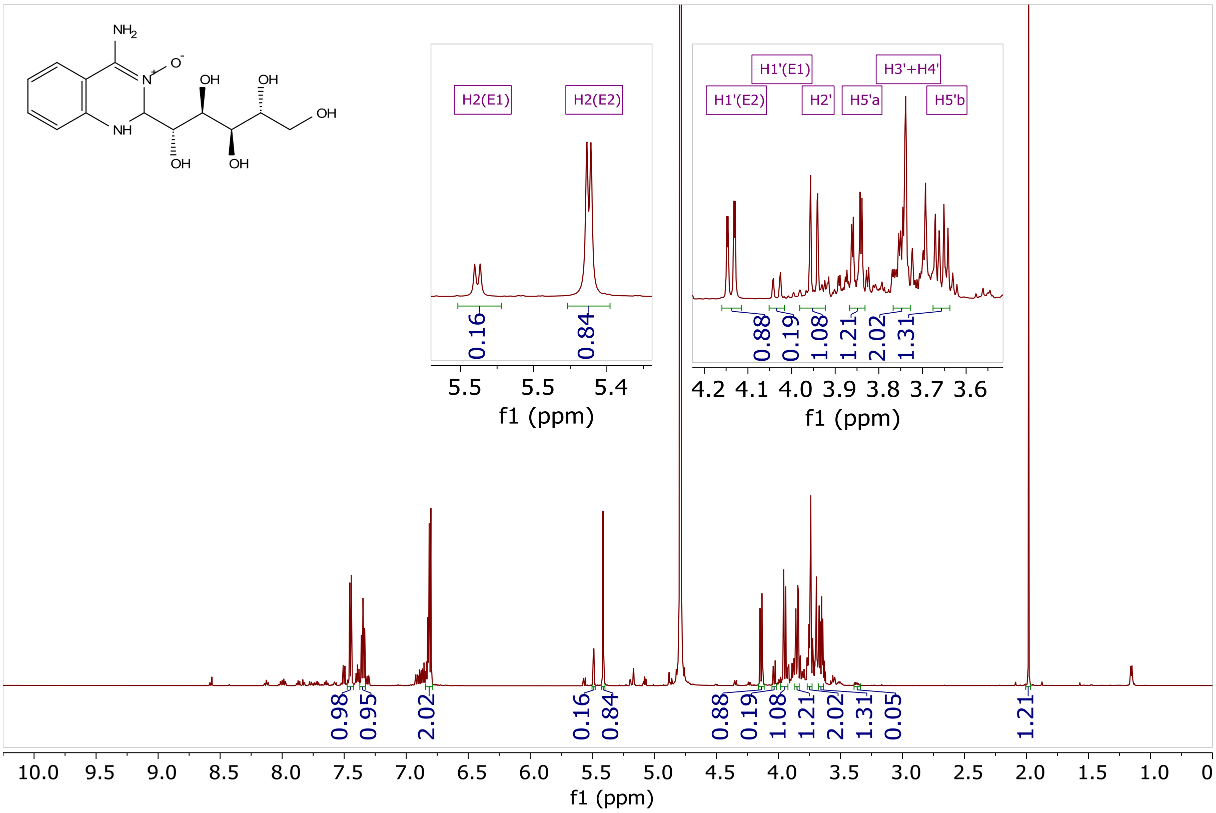

10.8 ^{13}C-NMR of the ABAO-adduct of mannose 15 (*erythro*)

Epimers were found in a ratio of ~15:85, where separated epimers' signals are assigned as (E1) and (E2). Residual amounts of acetate, mannose, and a decomposition product are present.

^{13}C NMR (151 MHz, D_2O) δ 149.9 (C4), 142.1 (C8a), 132.6 (C6/7), 122.3 (C5/8), 116.4, 112.9 (2x C5/C6/C7/C8), 105.3 (C4a), 71.3 (C2(E2)), 70.8 (C2(E1)), 68.8 (C3'/C4'), 68.3 (C1'), 67.0 (C3'/C4'), 65.7 (C2'), 61.2 (C5').

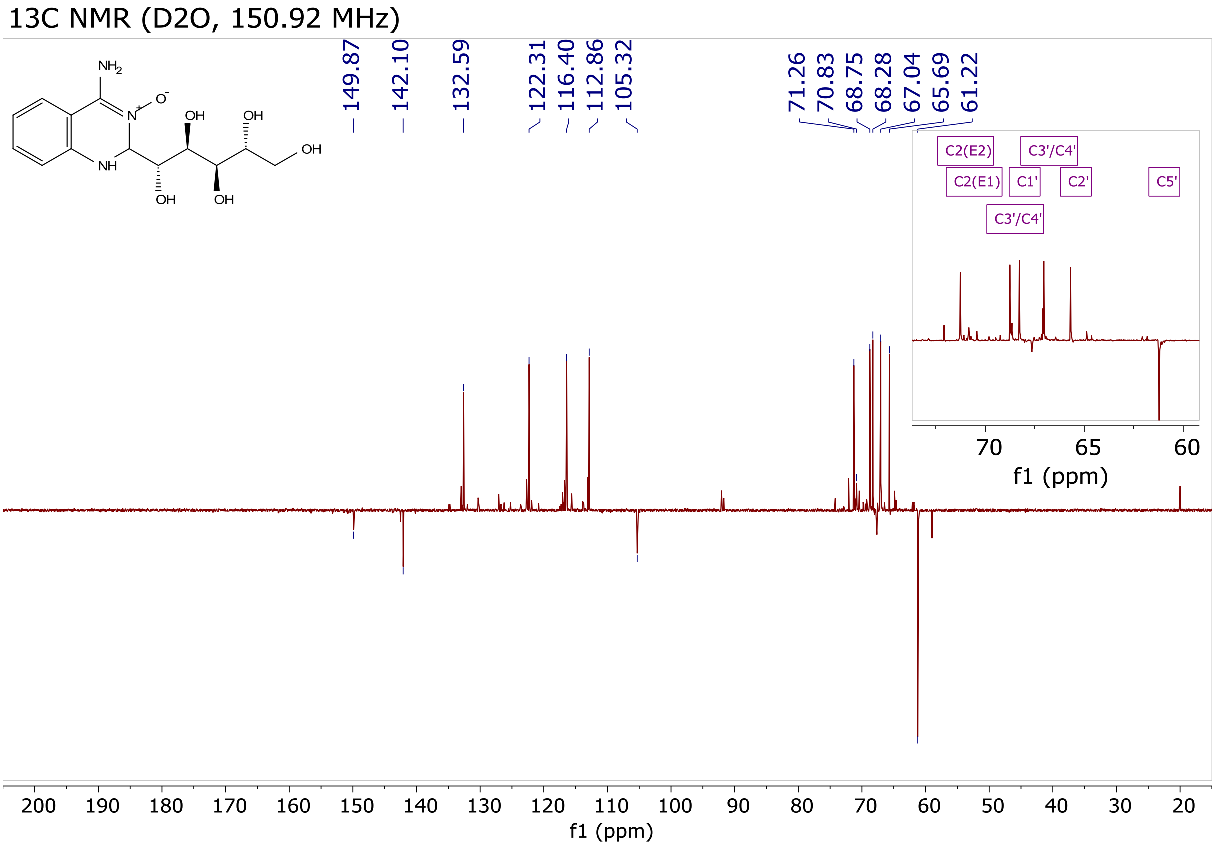

10.9 ^{1}H-NMR of the ABAO-adduct of threose 8 (*threo*)

Epimers were found in a ratio of ~35:64, where separated epimers' signals are assigned as (E1) and (E2). Excess ABAO, acetate from the buffer, and diethyl ether are still present.

^{1}H NMR (600 MHz, D_2O) δ 7.49 – 7.46 (m, 1H, H6/H7), 7.39 – 7.34 (m, 1H, H5/H8), 6.93 – 6.90 (m, 1H, H5/H6/H7/H8), 6.88 – 6.84 (m, 1H, H5/H6/H7/H8), 5.13 (d, *J* = 4.0 Hz, 0.35H, H2(E1)), 5.11 (d, *J* = 4.3 Hz, 0.7H, H2(E2)), 3.97 (t, *J* = 4.0 Hz, 0.65H, H1'(E2)), 3.86 (ddd, *J* = 6.6, 5.3, 3.6 Hz, 1H, H2'), 3.82 (dd, *J* = 4.0, 1.8 Hz, 0.3H, H1'(E1)), 3.65 (dd, *J* = 5.8, 1.7 Hz, 2H, H3').

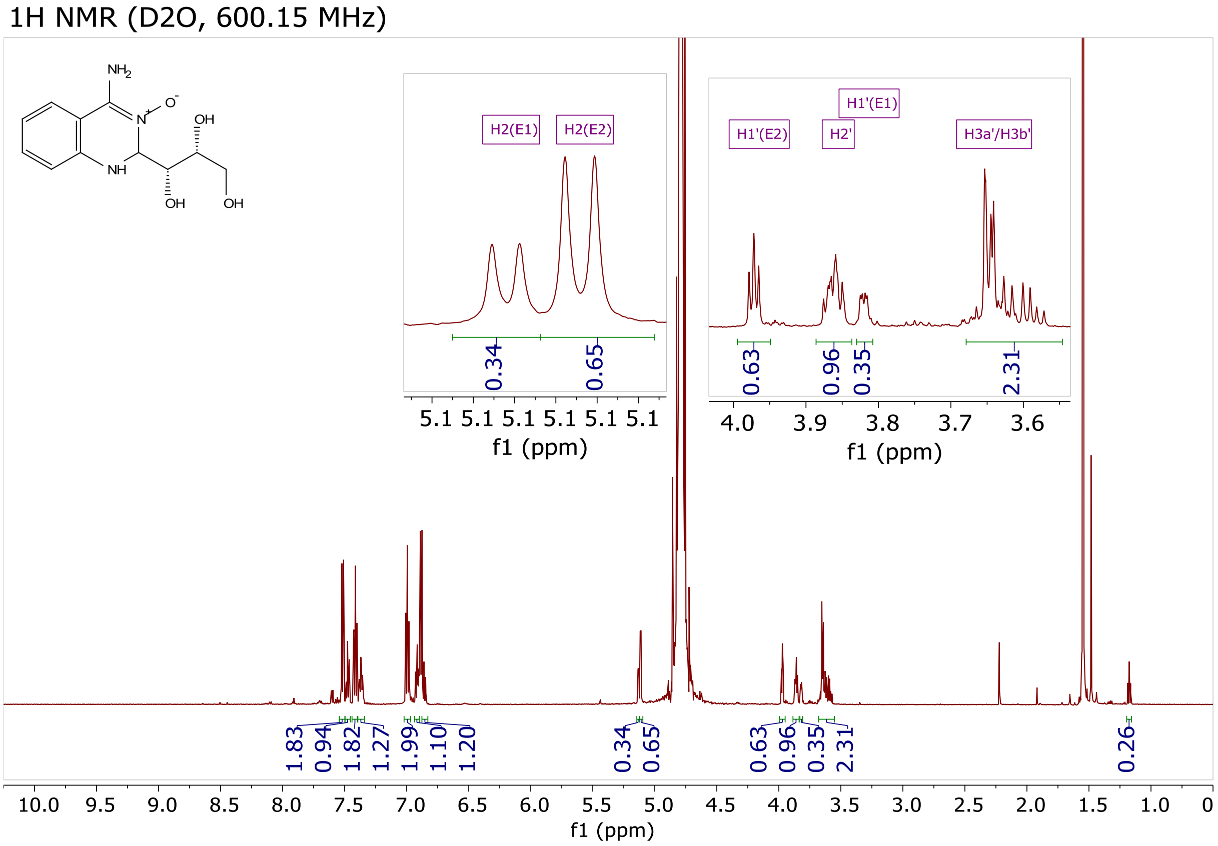

10.10 ^{13}C-NMR of the ABAO-adduct of threose 8 (*threo*)

Epimers were found in a ratio of ~35:64, where separated epimers' signals are assigned as (E1) and (E2). Excess ABAO, acetate from the buffer, and diethyl ether are still present.

^{13}C NMR (151 MHz, D_2O) δ 146.9 (C4), 142.2 (C8a), 133.5 (C6/C7), 124.0 (C5/C8), 119.2, 115.3 (2x C5/C6/C7/C8), 109.8 (C4a), 75.5 (C2(E2)), 74.5 (C2(E1)), 73.6 (C1'(E1)), 71.4 (C2'(E2)), 70.1 (C2'(E1)), 70.1 (C1'(E2)), 62.4 (C3').

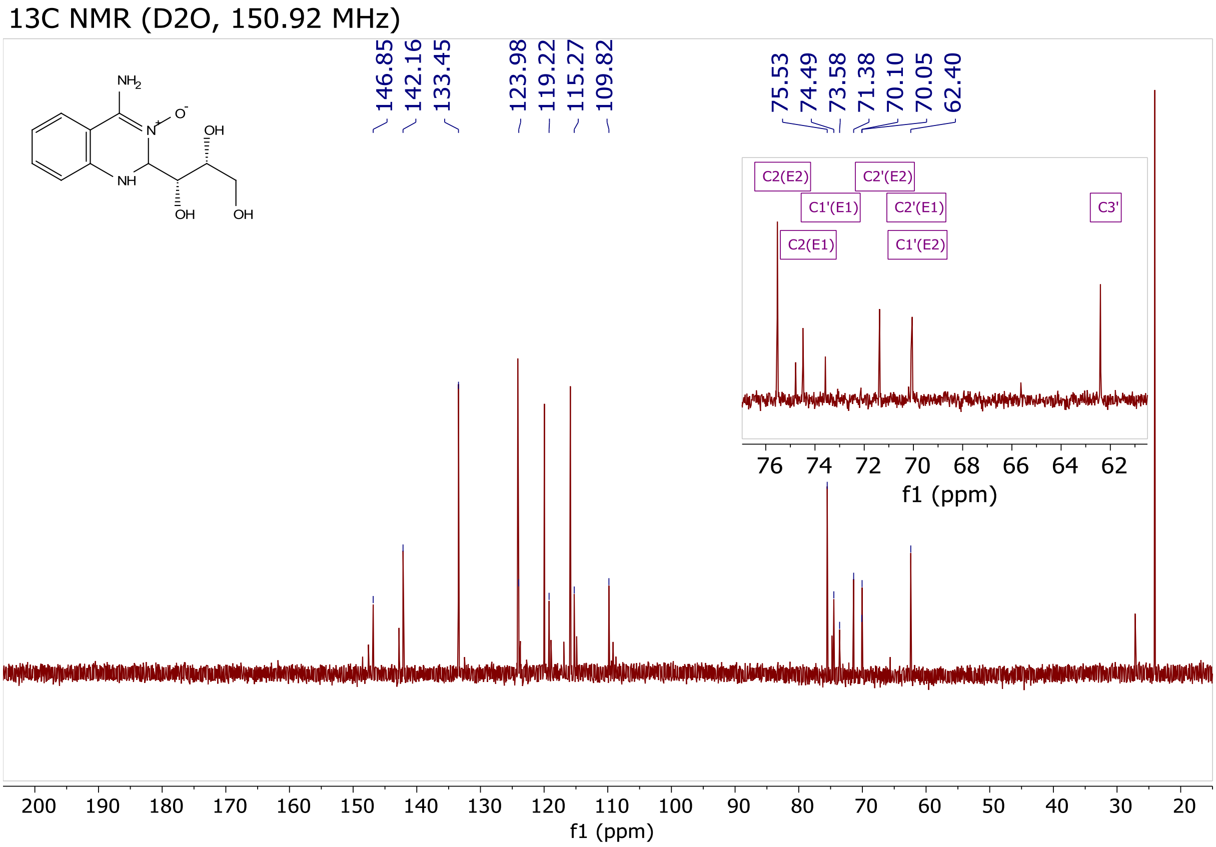

10.11 ^{1}H-NMR of the ABAO-adduct of arabinose 5 (*threo*)

Epimers were found in a ratio of ~40:60, where separated epimers' signals are assigned as (E1) and (E2). Residual amounts of acetate from the buffer are still present.

^{1}H NMR (400 MHz, $CDCl_3$) δ 7.49 (d, *J* = 8.0 Hz, 1H, H5/H8), 7.42 – 7.35 (m, 1H, H6/H7), 6.95 – 6.87 (m, 2H, H5/H8+H6/H7), 5.14 (d, *J* = 4.8 Hz, 1H, H2(E1)+H2(E2),), 4.13 (d, *J* = 5.0 Hz, 0.6H, H1'(E1),), 4.02 (d, *J* = 4.7 Hz, 0.4H, H1'(E2),), 3.86 – 3.78 (m, 1H, H4'a), 3.74 – 3.70 (m, 1H, H2'), 3.68 (d, *J* = 3.0 Hz, 1H, H3'), 3.65 – 3.60 (m, 1H, H4'b).

1H NMR (CDCl3, 400.13 MHz)

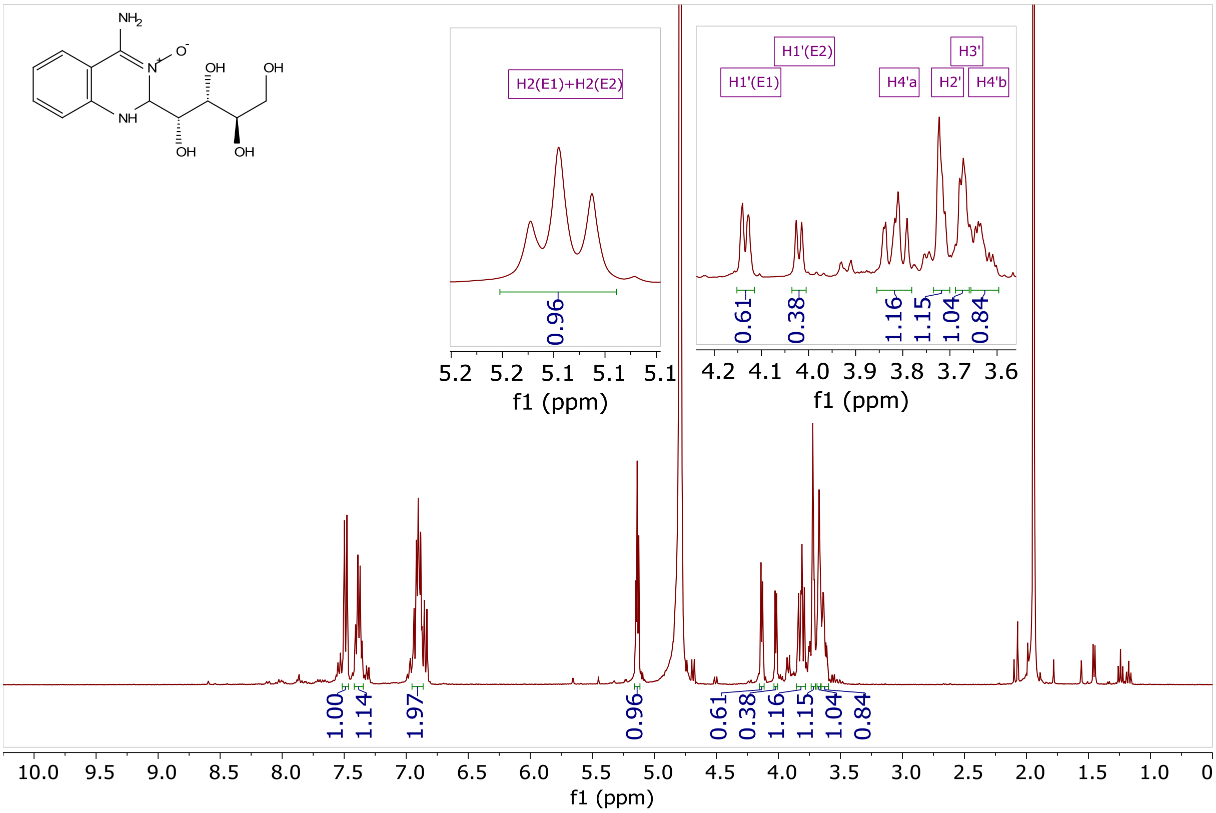

10.12 ^{13}C-NMR of the ABAO-adduct of arabinose 5 (*threo*)

Epimers were found in a ratio of ~40:60, where separated epimers' signals are assigned as (E1) and (E2). Residual amounts of acetate from the buffer are still present.

^{13}C NMR (101 MHz, $CDCl_3$) δ 149.0 (C4), 143.1 (C8a), 134.0 (C6/C7), 124.3 (C5/C8), 119.4, 119.0, 115.6, 115.2 (C5/C8+C6/C7), 108.7 (C4a), 74.6 (C2), 72.3 (C1'(E1)), 70.7 (C2'), 69.6 (C3'), 68.6 (C1'(E2)), 63.1 (C4').

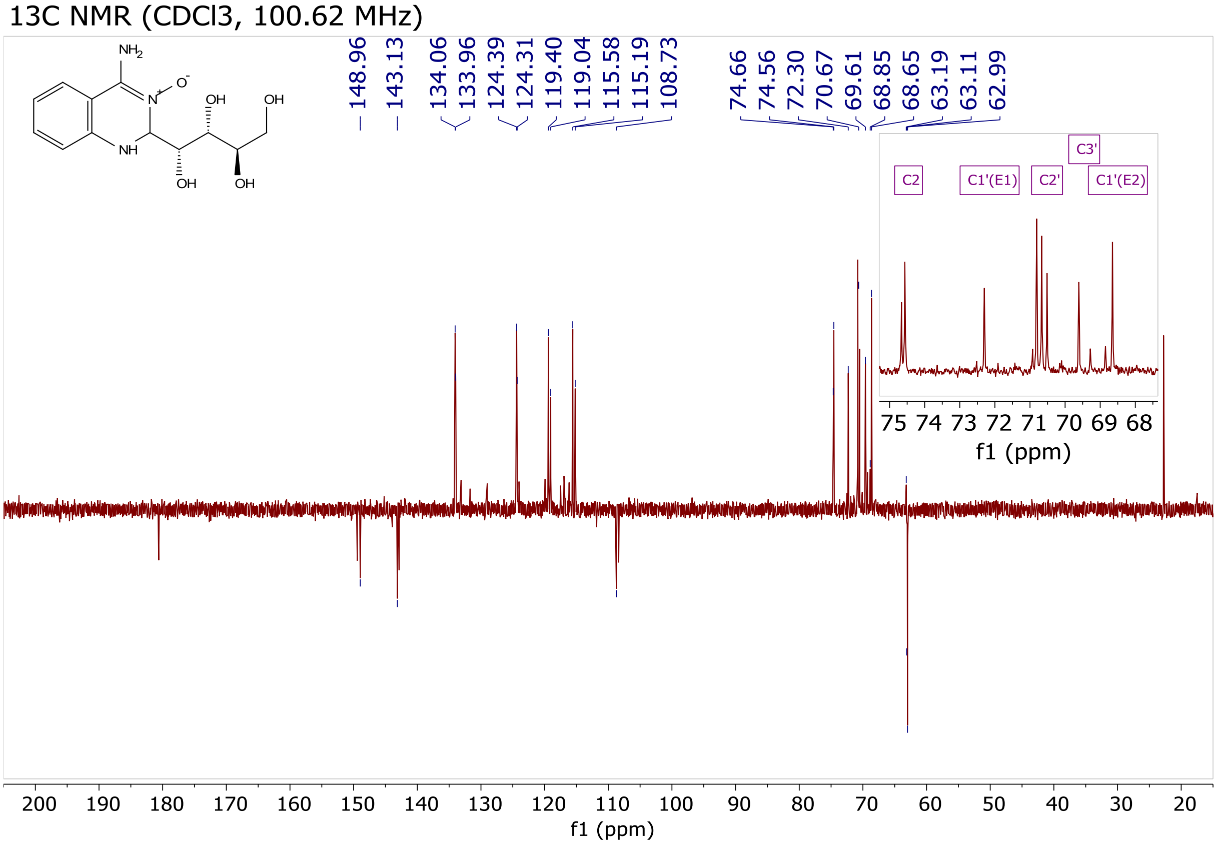

10.13 ^{1}H-NMR of the ABAO-adduct of xylose 11 (*threo*)

Epimers were found in a ratio of ~40:60, where separated epimers' signals are assigned as (E1) and (E2). Residual amounts of acetate from the buffer are still present.

^{1}H NMR (400 MHz, D_2O) δ 7.43 – 7.37 (m, 1H, H5/H8), 7.32 – 7.26 (m, 1H, H6/H7), 6.84 – 6.75 (m, 2H, H5/H8+H6/H7), 5.09 (d, *J* = 4.1 Hz, 1H, H2), 3.96 (t, *J* = 3.8 Hz, 1H, H1'(E1)), 3.79 (dd, *J* = 4.9, 2.0 Hz, 1H, H1'(E2)), 3.75 – 3.69 (m, 2H, H2'+H3'), 3.63 – 3.40 (m, 2H, H4').

1H NMR (D2O, 400.13 MHz)

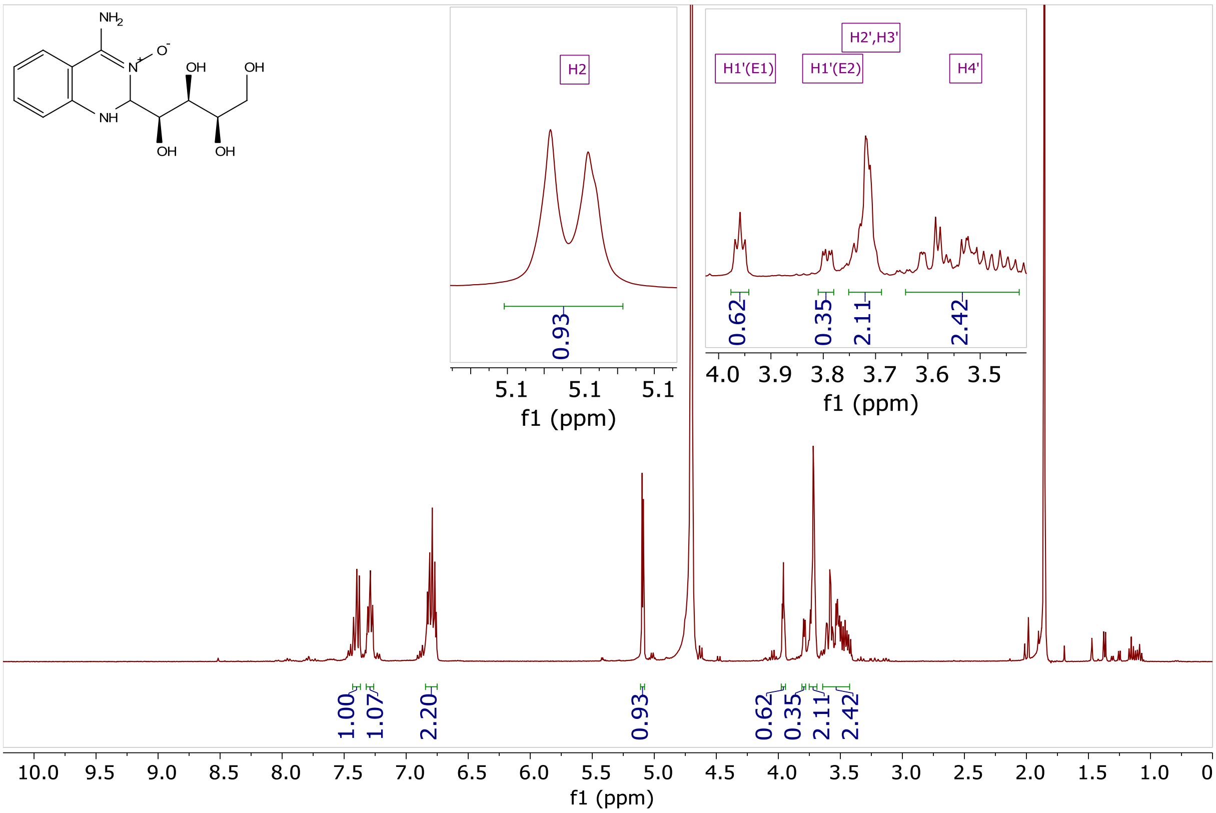

10.14 ^{13}C-NMR of the ABAO-adduct of xylose 11 (*threo*)

Epimers were found in a ratio of ~40:60, where separated epimers' signals are assigned as (E1) and (E2). Residual amounts of acetate from the buffer are still present.

^{13}C NMR (101 MHz, D_2O) δ 149.4 (C4(E1)), 148.9 (C4(E2)), 143.1 (C8a(E2)), 142.8 (C8a(E1)), 134.0 (C6/C7(E1)), 133.9 (C6/C7(E2)), 124.3 (C5/C8(E1)), 124.1 (C5/C8(E2)), 119.1 (C5/C6/C7/C8(E1)), 119.1, 115.2 (2x C5/C6/C7/C8(E2)), 115.2 (C5/C6/C7/C8(E1)), 108.5 (C4a(E2)), 108.3 (C4a(E1)), 74.0 (C2), 73.4 (C1'(E1)), 72.6 (C2'(E1)), 71.9 (C3'(E2)), 70.6 (C2'(E2)), 70.3 (C1'(E2)), 69.7 (C3'(E1)), 62.4 (C4'(E2)), 62.3 (C4'(E1)).

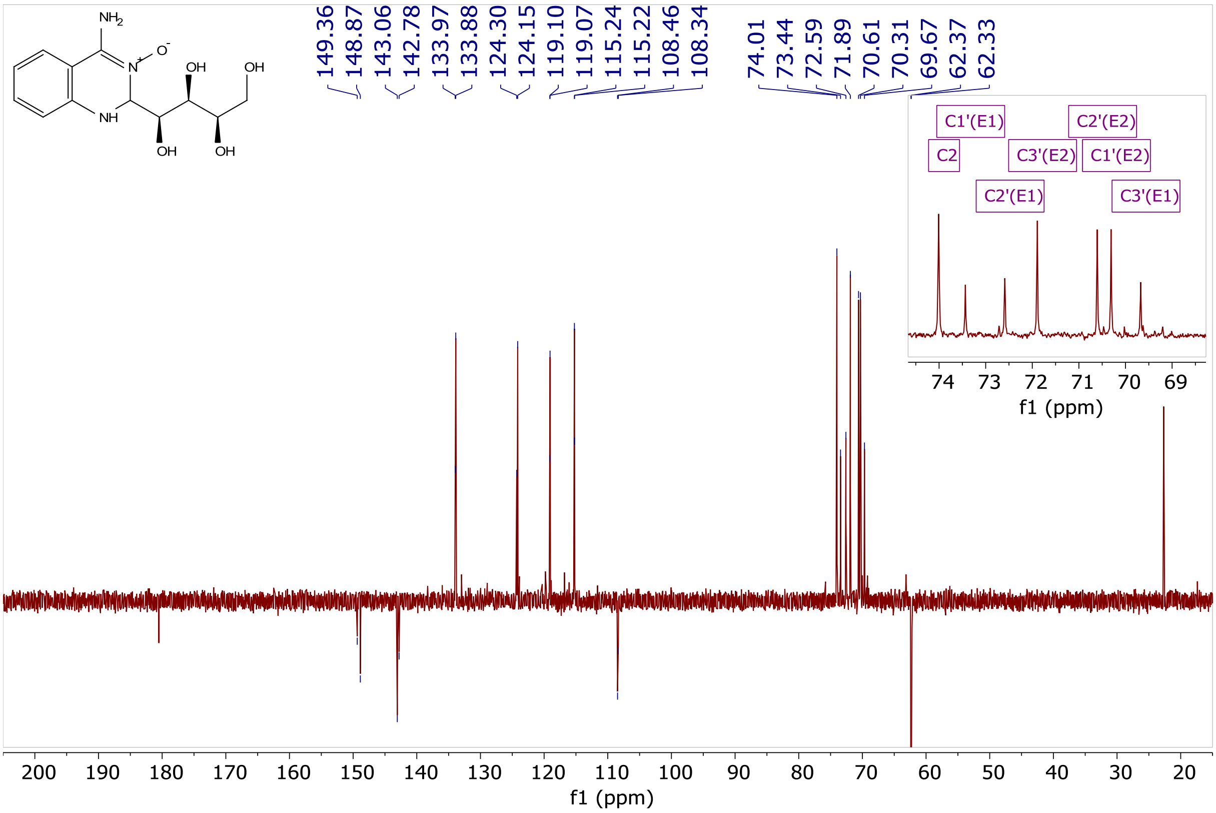

10.15 ^{1}H-NMR of the ABAO-adduct of galactose 6 (*threo*)

Epimers were found in a ratio of ~40:60, where separated epimers' signals are assigned as (E1) and (E2). Residual amounts of acetate from the buffer, residual galactose, and a decomposition product are present.

^{1}H NMR (600 MHz, D_2O) δ 7.51 (d, *J* = 8.0 Hz, 1H, H5/H8), 7.41 (td, *J* = 8.0, 7.4, 1.3 Hz, 1H, H6/H7), 6.98 – 6.83 (m, 2H, H5/H8+H6/H7), 5.17 (d, *J* = 5.8 Hz, 1H, H2), 4.15 (dd, *J* = 5.7, 1.7 Hz, 0.6H, H1'(E2)), 4.03 (d, *J* = 5.6 Hz, 0.4H, H1'(E1)), 3.98 – 3.93 (m, 1H, H4'), 3.84 (dd, *J* = 9.4, 1.8 Hz, 0.6H, H2'(E2)), 3.79 (d, *J* = 8.9 Hz, 0.4H, H2'(E1)), 3.66 – 3.62 (m, 3H, H3'+H5').

1H NMR (D2O, 600.15 MHz)

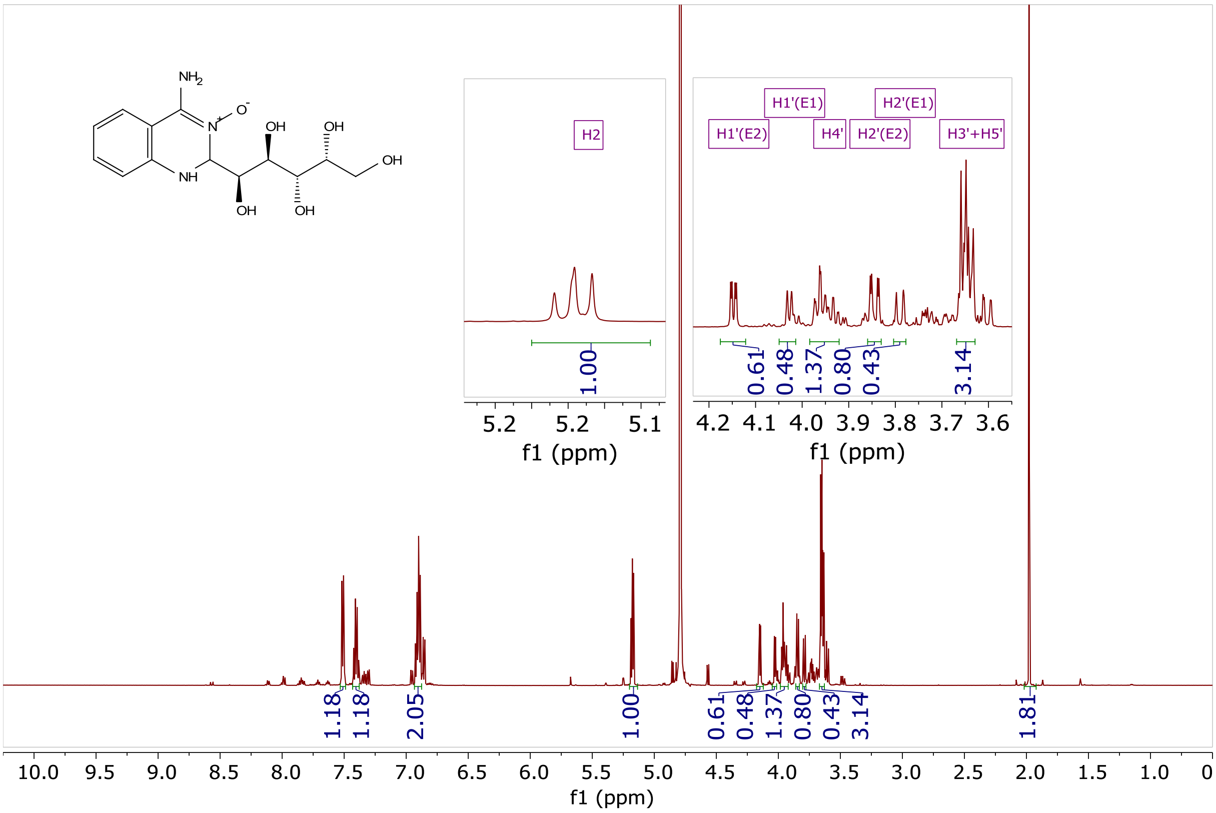

10.16 ^{13}C-NMR of the ABAO-adduct of galactose 6 (*threo*)

Epimers were found in a ratio of ~40:60, where separated epimers' signals are assigned as (E1) and (E2). Residual amounts of acetate from the buffer, residual galactose, and a decomposition product are present.

^{13}C NMR (151 MHz, D_2O) δ 150.8 (C4), 143.5 (C8a), 134.7 (C6/7), 124.7 (C5/8), 119.4, 115.7 (2x C5/C6/C7/C8), 107.9 (C4a), 74.1 (C2), 71.6 (C1'(E1)), 69.9 (C4'), 69.4 (C2'(E2)), 69.3 (C3'), 68.5 (C2'(E1)), 68.3 (C1'(E2)), 63.1 (C5').

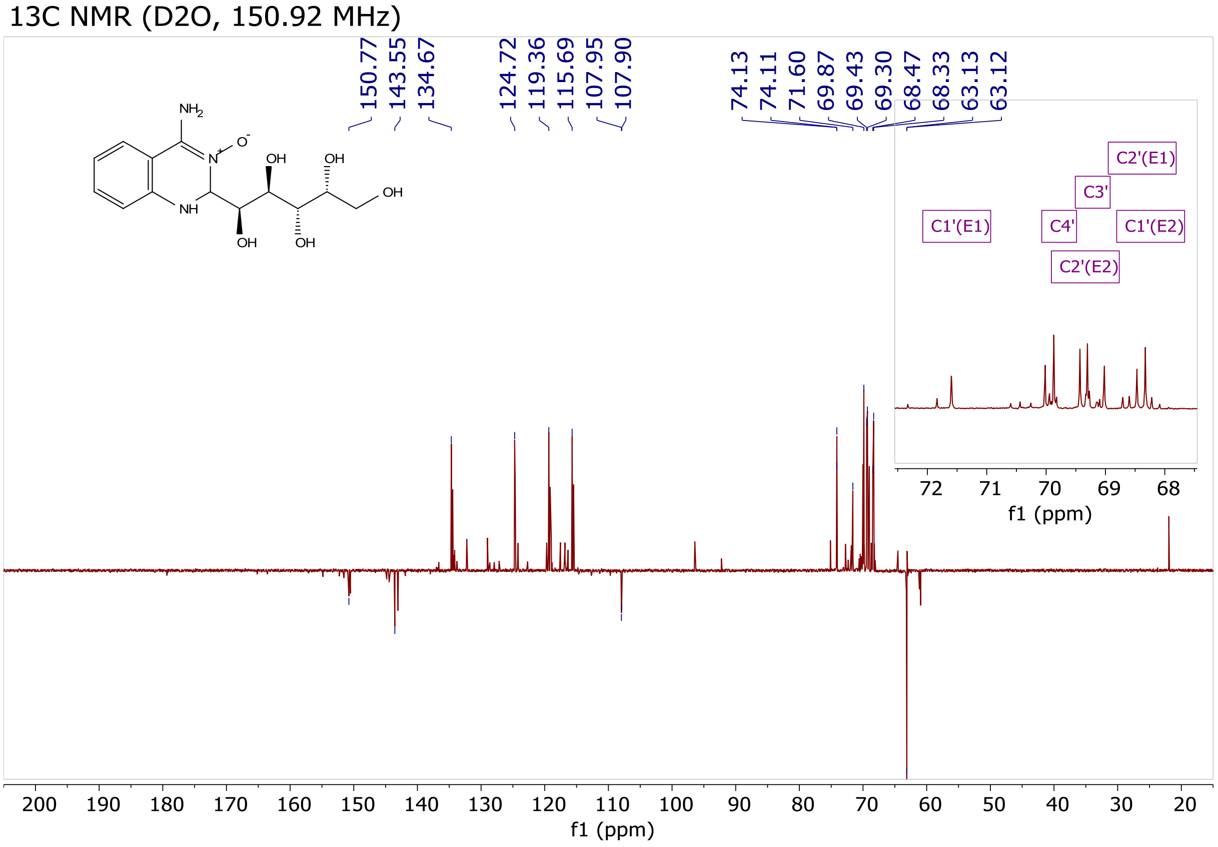

11 Sugars divided into *erythro*- and *threo*-families

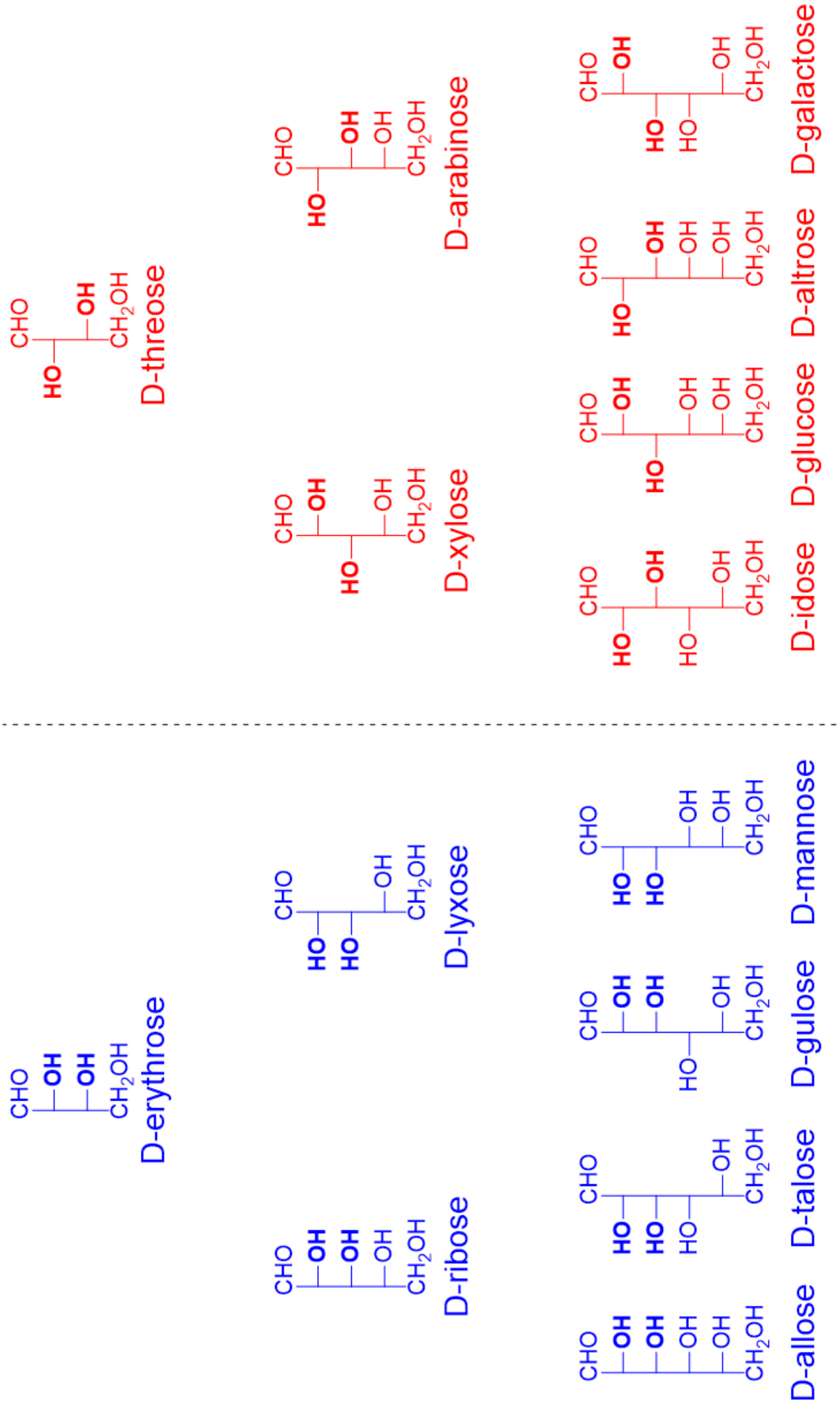

Figure S5: Standard sugars divided into the erythro- *and* threo-*family*

12 References

1. G. R. Fulmer, A. J. M. Miller, N. H. Sherden, H. E. Gottlieb, A. Nudelman, B. M. Stoltz, J. E. Bercaw and K. I. Goldberg, *Organometallics*, 2010, **29**, 2176-2179.
2. C. Stanetty and I. R. Baxendale, *Eur. J. Org. Chem.*, 2015, **2015**, 2718-2726.
3. A. Palmelund and R. Madsen, *J. Org. Chem.*, 2005, **70**, 8248-8251.
4. M. Draskovits, C. Stanetty, I. R. Baxendale and M. D. Mihovilovic, *J. Org. Chem.*, 2018, **83**, 2647-2659.
5. P. I. Kitov, D. F. Vinals, S. Ng, K. F. Tjhung and R. Derda, *J. Am. Chem. Soc.*, 2014, **136**, 8149-8152.
6. J. Pinnow and C. Sämann, *Ber. Dtsch. Chem. Ges.*, 1896, **29**, 623-632.
7. A. K. Ressmann, D. Schwendenwein, S. Leonhartsberger, M. D. Mihovilovic, U. T. Bornscheuer, M. Winkler and F. Rudroff, *Adv. Synth. Catal.*, 2019, **361**, 2538-2543.
8. P. E. Sorensen and W. P. Jencks, *J. Am. Chem. Soc.*, 1987, **109**, 4675-4690.
9. A. S. Serianni, E. L. Clark and R. Barker, *Carbohydr. Res.*, 1979, **72**, 79-91.
